# Light-enhanced liquid-phase exfoliation and current photoswitching in graphene–azobenzene composites

**DOI:** 10.1038/ncomms11090

**Published:** 2016-04-07

**Authors:** Markus Döbbelin, Artur Ciesielski, Sébastien Haar, Silvio Osella, Matteo Bruna, Andrea Minoia, Luca Grisanti, Thomas Mosciatti, Fanny Richard, Eko Adi Prasetyanto, Luisa De Cola, Vincenzo Palermo, Raffaello Mazzaro, Vittorio Morandi, Roberto Lazzaroni, Andrea C. Ferrari, David Beljonne, Paolo Samorì

**Affiliations:** 1 Nanochemistry Laboratory, ISIS & icFRC, Université de Strasbourg & CNRS, 8 allée Gaspard Monge, 67000 Strasbourg, France; 2 Laboratory for Chemistry of Novel Materials, University of Mons, Place du Parc 20, B-7000 Mons, Belgium; 3 Cambridge Graphene Centre, University of Cambridge, 9 JJ Thomson Avenue, Cambridge CB3 OFA, UK; 4 Laboratory of Supramolecular Biomaterials and Chemistry, ISIS & icFRC, Université de Strasbourg & CNRS, 8 allée Gaspard Monge, 67000 Strasbourg, France; 5 ISOF-CNR, via Gobetti 101, 40129 Bologna, Italy; 6 IMM-CNR Sezione di Bologna, via Gobetti, 101, 40129 Bologna, Italy; 7 Dipartimento di Chimica ‘G. Ciamician', Università di Bologna, via Selmi 2, 40126 Bologna, Italy

## Abstract

Multifunctional materials can be engineered by combining multiple chemical components, each conferring a well-defined function to the ensemble. Graphene is at the centre of an ever-growing research effort due to its combination of unique properties. Here we show that the large conformational change associated with the *trans*–*cis* photochemical isomerization of alkyl-substituted azobenzenes can be used to improve the efficiency of liquid-phase exfoliation of graphite, with the photochromic molecules acting as dispersion-stabilizing agents. We also demonstrate reversible photo-modulated current in two-terminal devices based on graphene–azobenzene composites. We assign this tuneable electrical characteristics to the intercalation of the azobenzene between adjacent graphene layers and the resulting increase in the interlayer distance on (photo)switching from the linear *trans*-form to the bulky *cis*-form of the photochromes. These findings pave the way to the development of new optically controlled memories for light-assisted programming and high-sensitive photosensors.

## Abstract (web summary)

Photochemical isomerisation can engender large conformational rearrangements, giving rise to switchable physical and electronic properties. Here, the authors use azo-benzene derivatives as addressable surfactants to facilitate the exfoliation of graphene and provide light activated modulation.

## Graphical abstract

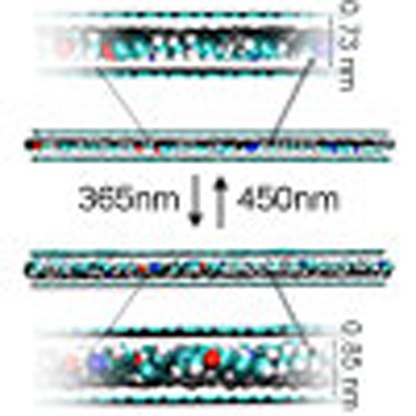

Graphene is a one-atom-thick two-dimensional material with unique mechanical, optical, thermal and electrical properties^1^, promising future emerging technologies, including flexible and wearable electronics^2^. Two main approaches are being followed for graphene production: bottom-up and top-down^3^. The former relies on the assembly of suitably designed molecular building blocks, undergoing chemical reaction to form covalently linked networks^4^. The latter occurs via exfoliation of graphite into graphene^3^,^5^. Bottom-up techniques, in particular those based on organic syntheses starting from small molecular modules^6^,^7^, when performed in liquid media, are both size limited, because macromolecules become less soluble with increasing size^6^, and suffer from the occurrence of side reactions with increasing molecular weight^6^. The growth on solid (ideally catalytically active) surfaces allows one to circumvent these issues. Substrate-based growth can also be achieved by chemical vapour deposition (CVD)^8^,^9^ or via silicon evaporation from silicon carbide^10^, which rely on the ability to follow a narrow thermodynamic path. Top-down approaches can be accomplished under different environmental conditions. Among them, liquid-phase exfoliation (LPE)^2^,^3^,^5^,^11^,^12^,^13^,^14^,^15^ is an attractive strategy, being extremely versatile and applicable to a variety of environments and on different substrates. While bottom-up methods, in particular CVD, can yield large size, LPE gives limited sheet sizes^11^,^16^,^17^,^18^. Nevertheless, LPE has several advantages. It is a viable low-cost process, which can be easily upscaled to mass-produced dispersions processable by well-established techniques such as spin-coating, drop-casting, screen-printing and ink-jet printing^17^. Increasing research efforts are being devoted to the production of graphene via LPE to improve the material's physicochemical and electrical properties.

Small organic molecules, such as surfactants or dispersion-stabilizing agents, can promote the exfoliation of graphite into graphene in organic solvents^5^,^11^, in particular when such molecules have higher adsorption energies on the basal plane of graphene than those involved in solvent–graphene interaction^11^. We recently found that long alkanes are particularly suitable for enhancing the yield of exfoliation^19^,^20^.

Photochromic molecules, in particular azobenzene-based molecules, covalently linked to reduced graphene oxide^21^, physisorbed directly on graphene^22^,^23^ or through pyrene anchoring groups^24^ on the graphene surface, or even when graphene is adsorbed on an azobenzene self-assembled monolayer^25^, can be used to reversibly modulate the graphene's electronic properties. Moreover, it was shown that covalent functionalization of gold surface^26^ or carbon nanotubes^27^ with azobenzene molecules can be used as a route for fabrication of light-driven electronic switches. Nonetheless, the use of additional responsive functions provided by a suitably designed molecule to assist the LPE of graphite into functional inks is still unexplored. This approach could lead to the production of functional hybrid materials and nanocomposites in a one-pot process.

Here we combine photochromic systems and LPE graphene exploiting the properties of these materials. We focus on a commercial alkoxy-substituted azobenzene, i.e., 4-(decyloxy)azobenzene. The presence of a long alkoxy side chain is expected to enhance the molecular interaction with graphene^28^, and promote the exfoliation of graphite towards graphene. Here we demonstrate that the use of 4-(decyloxy)azobenzene has two major advantages: first, the amount of graphene dispersed in *N*-methyl-2-pyrrolidone (NMP) can be enhanced by exploiting the photoisomerization of 4-(decyloxy)azobenzene molecules during exfoliation. Second, the isomerization of 4-(decyloxy)azobenzene from *trans* to *cis* and vice versa, when physisorbed between adjacent graphene layers leads to a reversible modulation of the inter-flake distance on the sub-Ångström scale. This is reflected in a light response of the electrical properties of the hybrid material.

## Results

### Liquid-phase exfoliation

**Figure 1:**
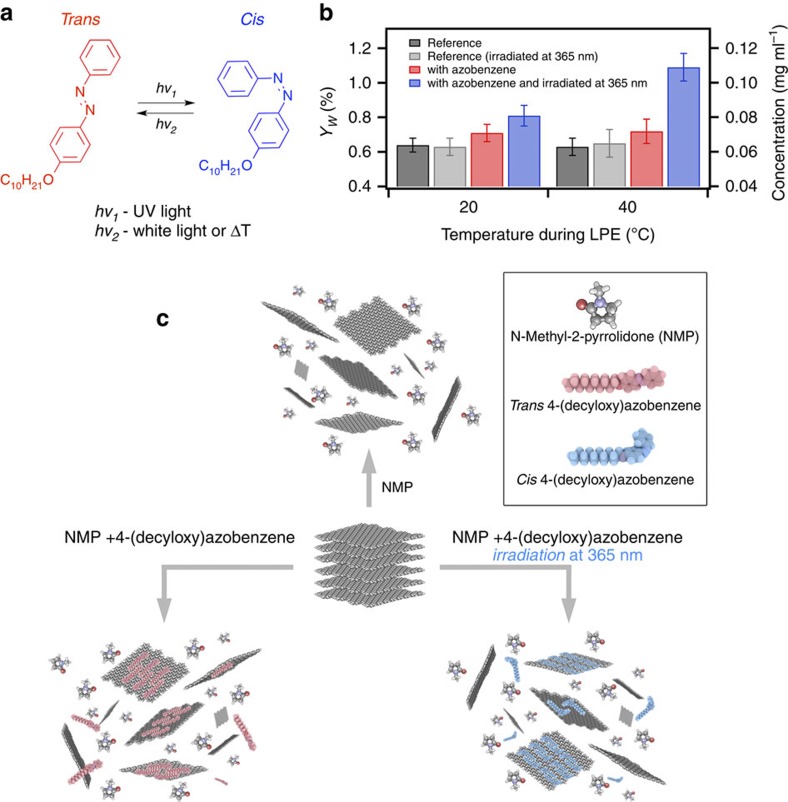
Light-assisted graphene's LPE process in the presence of 4-(decyloxy)azobenzene and yield of exfoliation. (**a**) Chemical structure of 4-(decyloxy)azobenzene and reversible *trans*–*cis* photoisomerization under UV and visible light. (**b**) *Y*_W_ and concentration after filtration. The error bars reflect a statistical analysis on 20 independent experiments. (**c**) Schematic representation of the LPE process under different experimental conditions: in NMP (reference experiment); and in NMP with azobenzene in dark and under UV irradiation.

To test the ability of alkoxy-substituted azobenzenes to increase *Y*_W_ (%), which is the yield of graphene exfoliation, defined as the ratio between the weight of dispersed graphitic material and that of the starting graphite flakes, as well as to exploit the photochromic nature of such molecules when interacting with graphene, vials containing NMP, graphite powder and azobenzene are exposed to tip sonication for 3 h (see Methods and Supplementary Note 1 for details). This experiment is carried out either in dark or under ultraviolet irradiation, using a portable ultraviolet lamp, at two temperatures: 20 and 40 °C (see experimental set-up in Supplementary Fig. 1). Subsequently, the dispersions are allowed to settle for 15 min, then decanted and centrifuged for 1 h at 10,000 r.p.m. Control samples are also prepared, consisting of dispersions prepared in the absence of 4-(decyloxy)azobenzene both in dark and irradiated at 365 nm at either 20 or 40 °C. To quantify the concentration of graphene after centrifugation, a mixture of graphene dispersion and 2-propanol (IPA) is first heated to 50 °C for 30 min and then passed through polytetrafluoroethylene membrane filters. The remaining solvent and weakly interacting 4-(decyloxy) azobenzene molecules are washed out several times with diethyl ether and IPA. Measurements of the filtered mass are performed on a microbalance to infer the concentration of graphitic material in dispersion, needed to quantify *Y*_W_ (%) (see Fig. 1b). The presence of adsorbed molecules may affect the mass measurements and *Y*_W_ (%). Thus, the heating step is necessary to completely desorb the 4-(decyloxy)azobenzene molecules, as proven with X-ray photoelectron spectroscopy (Supplementary Fig. 2 and Supplementary Note 2). By analysing 20 independent experiments, we find that when the exfoliation is performed in the absence of 4-(decyloxy)azobenzene (control experiments), comparable *Y*_W_ are observed when dispersions are kept at 20 °C (0.64%) or heated at 40 °C (0.62%). The irradiation at 365 nm does not affect *Y*_W_, neither at 20 nor at 40 °C (0.63% in both cases).

LPE in the presence of alkoxy-substituted azobenzene acting as dispersion-stabilizing agent can undergo different mechanisms depending on the isomeric form of the photochromic molecule (Fig. 1c). In our case, the presence of 4-(decyloxy)azobenzene has no major influence on *Y*_W_ of dispersions prepared in dark (0.71% and 0.72% at 20 and 40 °C, respectively). As previously reported^11^, the use of small organic molecules, in particular those based on aromatic cores^11^ and alkyl functionalization^19^,^20^, can promote graphite exfoliation because of their high affinity for the basal plane of graphene hindering interflake stacking. The relation between interaction energy and exfoliation yield is not straightforward. In some cases, molecules with lower adsorption energy on graphene give higher exfoliation yield, as demonstrated for graphite exfoliation^29^, as well as for exfoliation of a wide range of other layered materials^30^. This is because a critical step of the process is the adsorption of the molecule on the graphene surface and the displacement of the adsorbed solvent layer, overcoming an activation energy barrier that depends significantly on molecular structure and conformation. Therefore, even a minor increase of *Y*_W_ (0.06–0.09%) on addition of a small amount of 4-(decyloxy)azobenzene (5 wt%) highlights the importance of these molecules during LPE.

In contrast to the experiments performed in dark, irradiation with ultraviolet light at 365 nm increases the concentration of exfoliated material. At 20 °C we obtain ∼81±5 μg ml^−1^, corresponding to ∼30% increase in *Y*_W_, when compared with reference samples (63 μg ml^−1^). Yet, the largest increase in concentration is obtained by irradiating the dispersions heated at 40 °C, reaching 110±8 μg ml^−1^, which corresponds to a 75% increase in *Y*_W_. These results demonstrate that the addition of 4-(decyloxy)azobenzene during LPE leads to a significant increase in *Y*_W_ only in samples containing 4-(decyloxy)azobenzene irradiated at 365 nm. Molecules rapidly switching conformation, such as azobenzenes on ultraviolet (UV) illumination, heating and sonication, can overcome the activation barriers to adsorption, acting as ‘nano-spinners' that disrupt the adsorbed solvent layer more effectively than molecules having a static conformation. Therefore, the increase in concentration can be attributed to the motion resulting by the *trans*-to-*cis* photoisomerization. The *Y*_W_ temperature dependence may be due to differences in molecule–graphene interactions at 20 and 40 °C, as well as different kinetics of the *trans*-to-*cis* photoisomerization.

### *Trans*-to-*cis* photoisomerization and *cis*-to-*trans* relaxation

The transition from the thermodynamically stable *trans* to the metastable *cis* form can be induced by irradiation with UV light, and takes place on the picosecond timescale^31^,^32^, whereas the *cis*-to-*trans* isomerization is typically triggered by irradiation with visible light or heat^33^. The latter can also be activated by the use of ultrasound-induced mechanical force^33^. Ref. 34 claimed that mechanoisomerization of azobenzene typically results in the cleavage of the N=N azo bond. However, more recent theoretical calculations^35^ and experiments^36^ have challenged this view.

**Figure 2:**
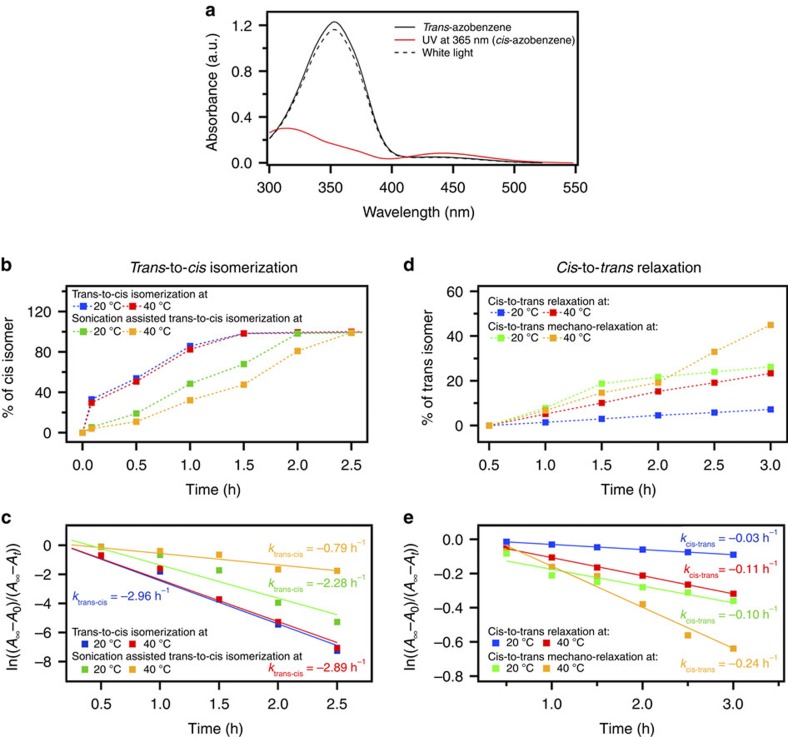
Spectroscopic characterization of *trans*-to-*cis* isomerization and *cis*-to-*trans* relaxation. (**a**) UV-ndash;visible spectra of 4-(decyloxy)azobenzene in NMP (1.62 mM) showing the disappearance of the 353-nm band and an increase of the 442-nm band for 365-nm irradiation. The solid black line corresponds to the parent *trans*-4-(decyloxy)azobenzene spectrum, the red line represents a typical spectrum of a solution irradiated with UV light (*cis*-4-(decyloxy)azobenzene), whereas the black dashed line corresponds to a *cis*-4-(decyloxy)azobenzene solution irradiated with visible light (thus re-transformed into *trans*-4-(decyloxy)azobenzene). (**b**) Conversion percentage from *trans* to *cis* as a function of UV irradiation time at 20 and 40 °C. (**c**) First-order kinetic plots and rate constants (*k*_*trans*–*cis*_) for *trans*-to-*cis* isomerization at 20 and 40 °C. (**d**) Conversion percentage from *cis* to *trans* as a function of time at 20 and 40 °C. (**e**) First-order kinetic plots and rate constants (*k*_*cis*–*trans*_) for the *cis*-to-*trans* relaxation at 20 and 40 °C. (**b**–**e**) Reference samples prepared without sonication: blue (20 °C) and red (40 °C); sonicated samples: green (20 °C) and orange (40 °C).

To gain a quantitative understanding of the photoisomerization of 4-(decyloxy)azobenzene, we perform a kinetic analysis of both *trans*-to-*cis* and *cis*-to-*trans* isomerization. The UV–visible spectrum of 4-(decyloxy)azobenzene dissolved in NMP (Fig. 2a, black line) shows an absorption maximum at 353 nm, arising from the *π*–π* transition^33^, and a peak at 442 nm, due to *n*–π* transitions^33^. The UV–visible spectrum of the above solution when irradiated at 365 nm shows a reduction of the *π*–π* transition band at 353 nm, and an increase in absorbance of the 442 nm (*n*–π* transition) peak, which is a characteristic feature of the *trans*-to-*cis* photoisomerization (Fig. 2a, red line; and Supplementary Fig. 3). The photostationary state can be reversibly converted back to the *trans* isomer on irradiation at 450 nm (see Methods for details). The yield of the conversion back to the thermodynamically stable *trans*-4-(decyloxy)azobenzene is higher than 95% (Fig. 2a, black dashed line).

To probe the *cis*-to-*trans* isomerization of the azobenzene molecules under experimental conditions corresponding to those used during the LPE process, i.e., at 20 and 40 °C, in the presence of ultrasound-induced mechanical forces, a stock solution of 4-(decyloxy)azobenzene in NMP (16.2 mM) is prepared, and then exposed to 365 nm light and sonicated for 3 h (see Supplementary Note 1 for details). Every 30 min (except for a first measurement performed after 5 min) 0.2 ml of UV-irradiated solution is transferred (in the dark) into a quartz cuvette, diluted 10 times with NMP. The optical absorption spectrum is then recorded (shown in Supplementary Fig. 4). Finally, to ensure the stability of the azo bond during sonication, a solution sonicated for 3 h is exposed to white light and an absorption spectrum is recorded. A similar procedure is applied for blank experiments, i.e., in the absence of ultrasound-induced mechanical forces.

Analysis of the absorption and proton nuclear magnetic resonance spectra (Supplementary Fig. 5) acquired for different experimental conditions allows us to quantify the percentage of conversion from *trans*- to *cis*-4-(decyloxy)azobenzene, as well as the rate constants for *trans*-to-*cis* isomerization. Both nuclear magnetic resonance and high-performance liquid chromatography data (Supplementary Figs 5 and 6, and Supplementary Note 3) show that the decyloxy chains are not being chopped from the azobenzene core. The slow kinetics of the *trans*-to-*cis* isomerization can be explained by the fact that both spectroscopic experiments and LPE process are carried out under unusual isomerization conditions, including a low UV light power density (0.34 mW cm^−2^). A different time (30 min) is needed for reaching a photostationary state, in the reference and sonicated samples, as displayed in Fig. 2b. Previous studies^36^,^37^ on the *trans*-to-*cis* isomerization of azobenzene in the presence of UV light showed that the reaction follows first-order kinetics and the rate constant *k* can be written as follows:


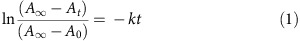


where *A*_0_, *A*_*t*_ and *A*_*∞*_ are the absorbances before irradiation, at irradiation time *t* and after irradiation for a prolonged time (ca. 6 h). Applying equation (1), we find that the isomerization constant *k*_*trans*–*cis*_ at 20 °C for the 4-(decyloxy)azobenzene linked to graphene is similar to *k*_*trans*–*cis*_ at 40 °C: 2.96 and 2.89 h^−1^, respectively. Significantly different *k*_*trans*–*cis*_ kinetics are observed for the samples exposed to ultrasound-induced mechanical forces, i.e., 2.28 and 0.79 h^−1^ for dispersions prepared at 20 and 40 °C, respectively. To ensure the stability of the azo bond during sonication, the two samples are exposed to white light, and the absorption spectra recorded. The absorption at 365 nm is consistent with the *trans*-4-(decyloxy)azobenzene spectra in both cases (Supplementary Figs 3 and 4), indicating that the molecules, in particular the azo bond, are stable during sonication at various temperatures.

Since the *trans*-to-*cis* isomerization and the LPE processes are performed under UV light, the possible *cis*-to-*trans* back-conversion can be considered promoted by thermal or mechano-thermal relaxation, for experiments performed in the absence or presence of ultrasound-induced forces, respectively.

To probe both thermal and mechano-thermal isomerization, four solutions (16.2 mM) are prepared in NMP and the UV–visible spectra recorded. The solutions are then irradiated with UV light (365 nm) for 3 h to photoisomerize the 4-(decyloxy)azobenzene from its *trans* to *cis* configuration (Supplementary Fig. 3). The solutions are then kept for 3 h at 20 and/or 40 °C, in the presence and/or absence of ultrasound-induced mechanical forces. Every 30 min, 0.2 ml of solution is transferred (in dark) onto a quartz cuvette and diluted 10 times with NMP. Absorption spectra are then recorded. Owing to the lack of white light during the 3 h relaxation, 4-(decyloxy)azobenzene molecules do not convert fully from their *cis*- to *trans*-isomers. As shown in Fig. 2c, the percentage of *cis*-to-*trans* conversion in the blank samples (without sonication) at 20 and 40 °C is ∼8.6% and 27.6%, respectively. In contrast, the ultrasound-induced conversion is much higher, i.e., 29.8% and 51.2% for samples kept at 20 and 40 °C, respectively. Similar to the *trans*-to-*cis* isomerization, the *cis*-to-*trans* relaxation of 4-(decyloxy)azobenzene follows first-order kinetics, and *k* can be obtained from equation (1). *k*_*cis–trans*_ for 4-(decyloxy)azobenzene kept at 20 °C is lower than *k*_*cis–trans*_ for 4-(decyloxy)azobenzene relaxed at 40 °C, i.e., 0.03 and 0.11 h^−1^, respectively (Fig. 2d). Notably different *k*_*cis–trans*_ are also observed for samples exposed to ultrasound-induced mechanical forces, i.e., 0.10 and 0.24 h^−1^ for dispersions kept at 20 and 40 °C, respectively.

These results indicate that the *trans*-to-*cis* isomerization of 4-(decyloxy)azobenzene strongly depends on the experimental conditions. In particular, it is hindered by ultrasound-induced mechanical forces, which most likely cause the occurrence of the competing *cis*-to-*trans* isomerization. Furthermore, comparison of *k*_*trans–cis*_ and *k*_*cis–trans*_ at 20 and 40 °C reveals that, at higher temperature, the process is more dynamic. As a result, the higher concentration of flakes (110 μg ml^−1^) obtained at 40 °C can be attributed to the dynamic conformational *trans*-to-*cis* and *cis*-to-*trans* changes of 4-(decyloxy)azobenzene molecules triggered simultaneously by the competing effect activated by UV light, temperature and ultrasounds.

### Analysis of dispersions

To fully characterize the exfoliated flakes, both qualitative and quantitative information are required. While quantitative insights can be assessed by providing the *Y*_W_, a qualitative analysis must give more relevant details, such as the percentage of single-layer graphene (SLG) and multi-layer graphene (MLG) flakes, the lateral size of the flakes and the presence/absence of defects. The number of graphene layers (*N*) can be quantified using high resolution-transmission electron microscopy (HR-TEM)^3^,^5^ and Raman spectroscopy^17^,^18^. Together with the information coming from electron diffraction patterns, in HR-TEM *N* can be directly counted by analysing the folded edges^38^. We focus on the samples prepared by LPE at 40 °C in the presence of azobenzene, while irradiating with UV light (highest increase in *Y*_W_), and compare them with those exfoliated in NMP at 40 °C under UV irradiation (in the absence of azobenzene). The statistical analyses of *N* and the lateral flake size are reported in Supplementary Fig. 7a,b, respectively (Supplementary Note 4). In terms of *N*, minor differences are observed between the reference sample and flakes exfoliated in the presence of azobenzene molecules, where the percentage of SLG amounts to 20% and 14%, respectively. The lateral size of the flakes is not affected by the presence of azobenzene molecules during LPE (Supplementary Fig. 7b). Representative HR-TEM micrographs are reported in Supplementary Figs 8, 9 and 10.

The Raman spectrum of graphite and MLG consists of two fundamentally different sets of peaks. Those, such as D, G and 2D, present also in SLG, due to in-plane vibrations^39^,^40^,^41^, and others such as the shear (C) modes^42^ and the layer-breathing modes (LBM)^40^,^43^, due to the relative motions of the planes themselves, are either perpendicular or parallel to their normal. The G peak corresponds to the high-frequency E_2g_ phonon at **Γ**. The D peak is due to the breathing modes of six-atom rings and requires a defect for its activation^41^,^44^,^45^. It comes from transverse optical phonons around the Brillouin Zone edge **K** (refs 41, 45), is active by double resonance^46^ and is strongly dispersive with excitation energy due to a Kohn anomaly at **K** (ref. 47). Double resonance can also happen as intra-valley process, i.e., connecting two points belonging to the same cone around **K** or **K**′. This gives the so-called D′ peak. The 2D peak is the D peak overtone while the 2D′ peak is the D′ overtone. Since 2D and 2D′ originate from a process where momentum conservation is satisfied by two phonons with opposite wave vectors, no defects are required for their activation, and are thus always present^38^. The 2D peak is composed of a single Lorentzian in SLG, whereas it splits into several components as *N* increases, reflecting the evolution of the electronic band structure^38^.

In disordered carbons, the G peak position, Pos(G), increases as the excitation wavelength decreases from infrared to ultraviolet^44^. Therefore, the dispersion of the G peak, Disp(G), i.e. the rate of change of Pos(G) with the laser excitation wavelength, increases with disorder^44^. Similar to Disp(G), also the full width at half maximum of the G peak, FWHM(G), always increases with disorder^44^. The analysis of the intensity ratio of the D and G peaks, *I*(D)/*I*(G), combined with that of FWHM(G) and Disp(G) allows us to discriminate between disorder localized at the edges and disorder in the bulk of the samples. In the latter case, a higher *I*(D)/*I*(G) would correspond to higher FWHM(G) and Disp(G).

**Figure 3:**
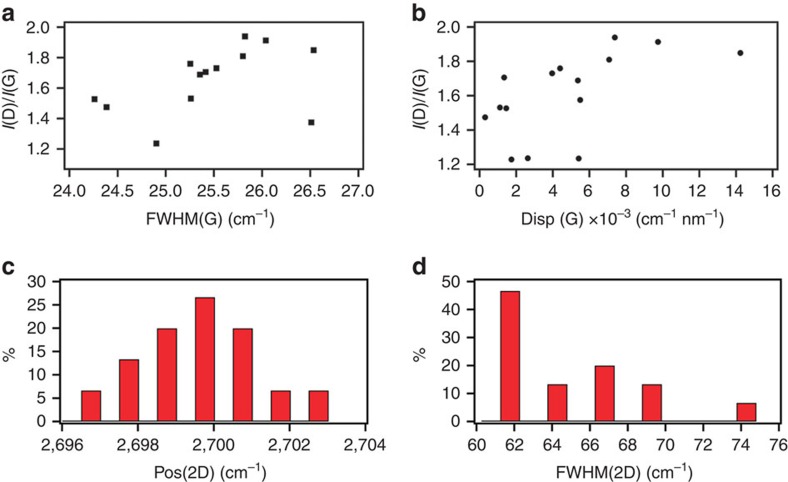
Raman analysis of graphene/4-(decyloxy)azobenzene films. *I*(D)/*I*(G) as a function of (**a**) FWHM(G) and (**b**) Disp(G). Distribution of (**c**) Pos(2D) and (**d**) FWGM(2D).

Raman measurements are done on the same set of samples to characterize the quality of the graphitic material. Figure 3a,b show a small correlation between these parameters. This implies that the D peak is mostly due to edges, as well as the presence of some defects in the samples. FWHM(2D) (Fig. 3d) is larger with respect to that of graphene flakes produced by micromechanical cleavage, but still has a single Lorentzian line shape. This is consistent with the presence of MLGs composed of folded or juxtaposed SLGs, but still electronically decoupled^17^.

**Figure 4:**
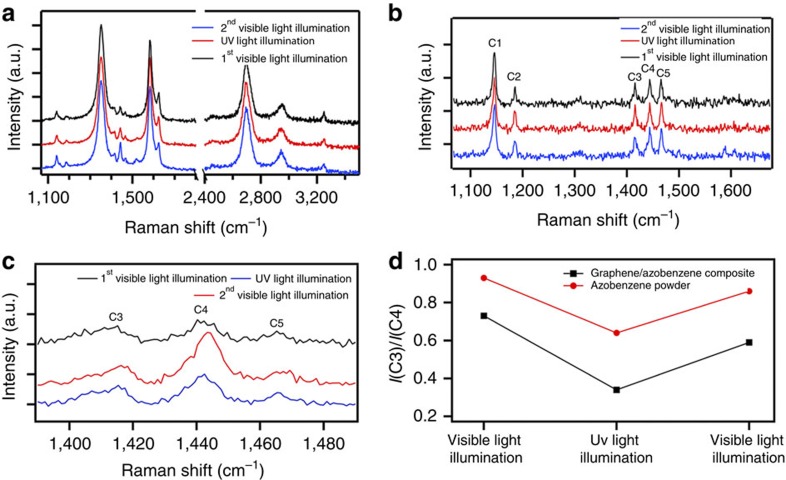
Evolution of Raman spectra on illumination. (**a**) Comparison of the Raman spectrum of a graphene/4-(decyloxy) azobenzene hybrid film after 90-min illumination with visible light, UV light and visible light again to probe the effect of isomerization. (**b**) The same experiment on azobenzene powder. (**c**) 4-(decyloxy)azobenzene Raman peaks C3, C4 and C5 extracted from graphs in **a** showing the change of peaks ratio on illumination, which confirms the isomerization. (**d**) Dependence upon illumination of *I*(C3)/*I*(C4) for both hybrid material and powders.

Figure 4a plots the Raman spectra of a graphene–azobenzene film after visible and UV light illumination, acquired at 514.5 nm with power <250 μW to avoid any Raman laser-induced isomerization of azobenzene. None of the graphene Raman parameters show significant change on UV or visible light irradiation indicating negligible change in the defects and doping.

The *trans*-to-*cis* isomerization can also be monitored by Raman spectroscopy^24^,^48^. Figure 4b plots the Raman spectrum of azobenzene after both visible and UV light illumination. The spectrum is composed of five main peaks, labelled C1–C5 (ref. 48). These can also be seen on the spectra of the graphene–azobenzene hybrids (Fig. 4c). The intensity ratio of peaks C3 and C4 can be used to monitor the isomerization of azobenzene as described in ref. 48. On illumination with visible light, the photochromic molecules fall into the *trans* configuration and *I*(C3)/*I*(C4) approaches ∼1. When the sample is illuminated with UV light, azobenzene molecules undergo *trans*-to-*cis* isomerization and *I*(C3)/*I*(C4) decreases markedly. The effect is fully reversible and the *trans* form is recovered by a further visible light exposure as shown in Fig. 4d.

### X-ray analysis

Powder X-ray diffraction is further used to characterize the structure of the graphene–azobenzene hybrid powder and to compare it with the control samples (Supplementary Note 5). Similarly to graphite, the powder prepared from LPE has a sharp peak at 26.7°, corresponding to an interlayer spacing of ∼0.33 nm (Supplementary Fig. 11a). The X-ray diffraction patterns of powders prepared from graphene/*trans*-azobenzene and graphene/*cis*-azobenzene show new peaks at 2*θ*=12.3° (*d*_spacing_∼0.72 nm) and 9.9° (*d*_spacing_∼0.89 nm), respectively (Supplementary Fig. 11b,c). This confirms that *trans* and/or *cis*-azobenzenes can sandwich between graphene layers, thus increasing the overall spacing.

### Electrical characterization

To probe the electrical properties of the graphene–azobenzene hybrid and, in particular, to explore the potential light-responsive nature of the material, we drop-cast a ∼100-nm-thick graphene–azobenzene film on a n^++^Si/SiO_2_ substrates exposing pre-patterned interdigitated gold electrodes (channel length=10 μm; Fig. 6a,b).

**Figure 5:**
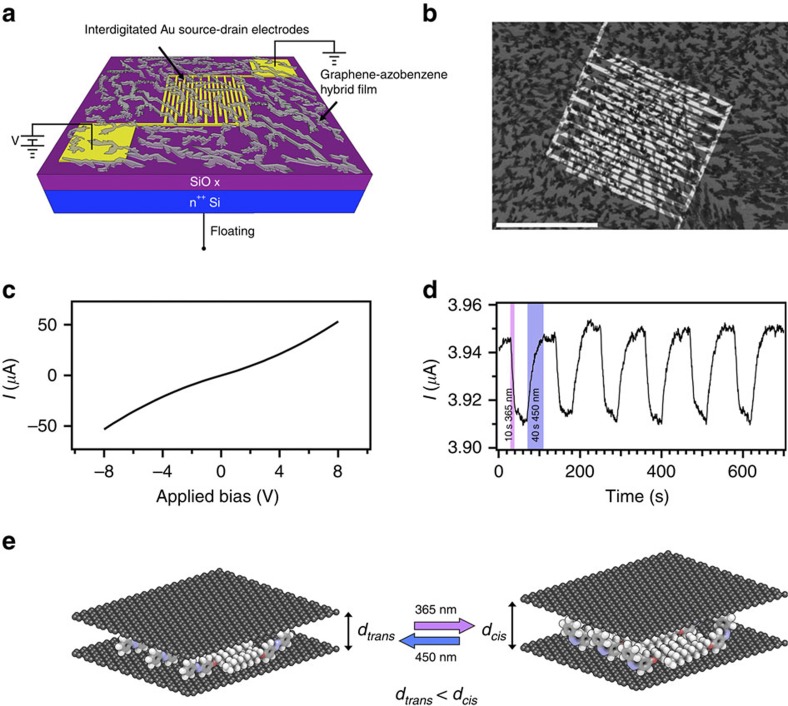
Electrical characteristics of hybrid materials. (**a**) Scheme of the two-terminal device configuration, (**b**) scanning electron microscopy image of interdigitated Au electrodes covered with a hybrid film. Scale bar, 500 μm. (**c**) *I*–*V* characteristics, (**d**) optical modulation of current response for a static bias and dynamic alternative UV and visible light irradiation cycles. (**e**) Schematic of graphene-azobenzene hybrid undergoing UV and visible irradiation cycles.

**Figure 6:**
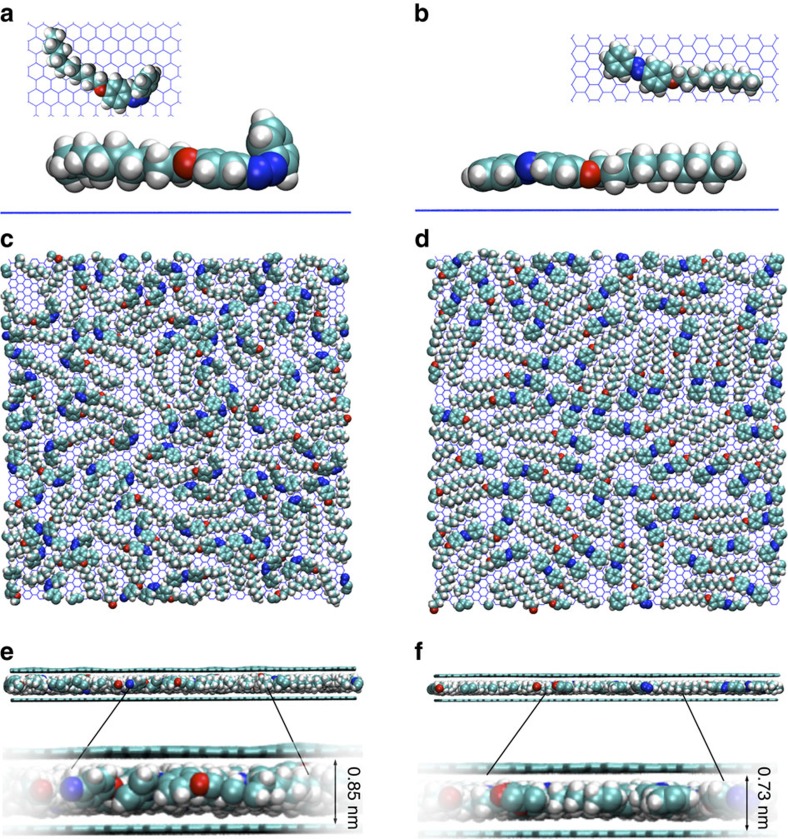
Molecular modelling of graphene interaction with *cis* and *trans* azobenzene. Side and top views of a single (**a**) *cis*- and (**b**) *trans*-azobenzene molecule adsorbed on SLG. Top view of the (**c**) *cis* and (**d**) *trans*-monolayer, and side view of the molecular system consisting of the (**e**) *cis* and (**f**) *trans*-monolayer sandwiched between two SLGs, with the indication of the SLGs separation.

Typical *I*–*V* curves exhibit a nonlinear resistive behaviour, as shown in Fig. 5c. The conductivity of the graphene–azobenzene hybrid is lower than that measured in control devices, i.e., comprising as active layer graphene exfoliated in NMP in the absence of azobenzene (Supplementary Fig. 12). Figure 6d shows the current modulation in a two-terminal device with graphene–azobenzene on cycles of UV and visible light irradiation, for 1 V applied between the two electrodes. One cycle consists of 10-s UV light (365 nm), 1-min rest in dark followed by 40-s visible light (450 nm) irradiation. Under UV exposure (magenta background) a current decrease is detected, which reversibly increases to about the initial value under visible light irradiation (violet background) over various cycles. No fatigue is observed during six cycles of photoisomerization. Conversely, the control devices show no response to light (Supplementary Fig. 12). Noteworthy, in some cases the increase of current in the hybrid films can be the result of *π*-electron interactions between the molecules and graphene^49^; however, such phenomenon, known as increased photoconductivity, is irreversible and can only be modulated by varying the ratio between graphene and *ad hoc* molecules.

In the azobenzene–graphene film (Supplementary Figs 13 and 14), the molecules are physisorbed on top and in between the graphene sheets. Under UV irradiation, the azobenzene molecules undergo a conformational change from the less-bulky *trans* isomer to the bulkier *cis* isomer. Such a process is therefore accompanied by an increase in the few-layer graphene (FLG) inter-sheet distance, thereby hindering the charge transport via hopping between the sheets, resulting in lower conductivity of the hybrid film. The conductivity of the hybrid material can be fully restored by irradiating with visible light, causing the *cis*-to-*trans* photoisomerization.

To gain greater insight into the conduction mechanism and the effect of isomerization, we then perform Kelvin probe measurements (Supplementary Note 6). We find that the work function is reversibly photo-modulated over various cycles (Supplementary Fig. 15), providing further evidence for the isomerization of the azobenzene molecules. This difference in work function has a direct effect on charge injection from the electrodes to the graphene/azobenzene conductive material, which is less favourable in the presence of the *cis* isomer. Light modulation can be applied multiple times to the systems, with no evidence of decrease in performance. We assign this to graphene's shielding of the azobenzene molecules from the outer environment. An ideal case consists of stacks of alternating graphene and azobenzenes, the latter having a thickness, which can be photo-modulated (Fig. 5e). In reality, the flakes (with average lateral sizes of 200 nm) form aggregates possessing a poor degree of order. Within these aggregates the azobenzenes are intercalated in between adjacent layers of graphene. The control samples show no change in work function on UV and visible light cycling (Supplementary Fig. 15).

### Molecular modelling simulations

To gain a better understanding of the interactions of the azobenzene molecules with graphene, we perform molecular dynamics simulations (see Supplementary Note 7 for details). All calculations are done using the Groningen Machine for Chemical Simulations (GROMACS)^50^ package and a modified version of the all-atom Optimized Potentials for Liquid Simulations (OPLSAA) force field developed for azobenzenes^51^,^52^. To assess the relative adsorption affinity of the *trans* versus *cis* conformations, we first compute the interaction energy for a single azobenzene molecule by running a 1-ns simulation in vacuum. The graphene layer is taken as an infinite rigid body in these calculations. The computed interaction energies of −130.1±7.5 and −115.7±8.0 kJ mol^−1^ for *trans*- and *cis*-azobenzene, respectively, are consistent with the expected stronger adsorption of *trans*-azobenzene, resulting from its planar geometry and the concomitant maximized *π*–*π* interactions between the phenyl groups and graphene. The average adsorption distance for the *trans* molecule is ∼0.07 nm shorter than *cis* (0.34 versus 0.41 nm). Figure 6a,b plots top and side views of the adsorbed *cis* and *trans* molecules from the last snapshots along the molecular dynamics trajectories.

It should be noted that the conformation giving the higher exfoliation yield is the *cis* one, with lower interaction energy with graphene (14.4 kJ mol^−1^ difference). This counterintuitive finding highlights the importance of kinetic factors on exfoliation yield rather than thermodynamic factors, in good agreement with previous results obtained with rigid molecules^29^,^30^. The 70% increase in exfoliation yield could be due to the rapid, dynamic change of conformation between *cis* and *trans* that the molecules undergo during the exfoliation, which is more rapid at high temperature and under sonication (Fig. 2).

We then study the supramolecular organization of *trans*- and *cis*-azobenzenes on SLG in vacuum. In the absence of experimental structural data, we consider highly regular self-assembled monolayers (Supplementary Fig. 16) and test their stability by performing simulations of the assemblies at room temperature. The regular assemblies are unstable at 300 K and disassemble after few tens of picoseconds, suggesting that the azobenzene molecules form disordered layers at the SLG surface. To investigate the formation and structure of such disordered layers, we use the following protocol. Azobenzene molecules are introduced one by one in the simulation box at a distance of 1.8 nm above the plane of the infinite SLG with a random orientation. The initial atomic velocities are assigned so that the resulting vectors point towards the SLG, thereby prompting physical adsorption on the surface. Room-temperature molecular dynamics simulations of a few hundreds of picoseconds are then performed to explore the configurational space after each molecular deposition, and the last saved configuration is used as input for the landing of the subsequent molecule. The scheme is iterated multiple times until the desired surface coverage is reached. A 5-ns simulation is then performed on the final structure to equilibrate the monolayer at room temperature. A more detailed description of the methodology is provided in Supplementary Note 8. Full coverage of the graphene periodic layer of 115.8 nm^2^ is reached when 64 (72) molecules of *trans* (*cis*)-azobenzene are adsorbed, which translates into a surface contact area on graphene of ∼1.8 nm^2^ for the *trans* and ∼1.6 nm^2^ for the *cis* conformation. Figure 6c,d plots a top view of the azobenzene monolayers obtained for the two conformers in the case of full coverage. Supplementary Figs 17 and 18 show the formation of those monolayers as a function of the number of molecules adsorbed on SLG.

To validate the hypothesis that the insertion of a *trans*-/*cis*-monolayer sandwiched between two SLGs can result in different interlayer spacing according to the ideal model, a second SLG is placed on top of the azobenzene monolayers prepared using the deposition protocol described above. The 1-ns simulations are then done at room temperature. The results show that *cis*-azobenzenes maintain the SLG at a distance ∼0.85 nm, while the layer separation decreases to ∼0.73 nm for *trans* (Fig. 6e,f), in excellent agreement with the X-ray data. This supports the view that photoisomerization of the molecules from *trans* to *cis* can favour the exfoliation process. To investigate the effect of this switchable separation on charge transport, we perform quantum chemical calculations (at the intermediate neglect of differential overlap level^53^) on two square-shaped nanographenes (∼20 nm^2^) interacting either through-space or in the presence of the sandwiched (*trans* or *cis*) azobenzenes (Supplementary Fig. 19). In the first case (no sandwiched layer), the electronic couplings mediating positive and negative charge carrier tunnelling from one nanographene to the other, as obtained from the diabatization scheme reported in ref. 54, decrease by a factor ∼100 when the interlayer distance is increased from 0.73 (corresponding to the azobenzenes in *trans* conformation) to 0.85 nm (*cis* conformation). In both cases, these interactions are very small due to the fast fall-off decay of the nanographene wavefunction overlap. Interestingly, the corresponding couplings calculated in the presence of the sandwiched azobenzenes are several orders of magnitude larger and show a less pronounced change (by a factor ∼10) on photoisomerization, suggesting that the loss in electrical transmission associated with the larger inter-graphene distances is at least partly compensated by conformational-dependent superexchange effects involving the molecular orbitals of the azobenzenes. Our experimental current modulation on photoisomerization of azobenzenes (Fig. 5d) is much smaller than the calculated one (factor ∼100). This can be understood considering that the simulations are run for two SLG intercalated by a tightly packed, physisorbed, self-assembled layer of azobenzene, whereas the experiments are done for hybrid films with a distribution of layer thicknesses (Supplementary Fig. 7a) separated by a small quantity of azobenzene (below 5% of the area of graphene).

As aforementioned the ideal model does not fully describe our real system that consists of poorly ordered aggregates comprising flakes intercalated with azobenzenes, and charges hopping between adjacent graphenes. The distance between the adjacent graphene sheets can be varied on photoisomerization of the azobenzenes.

## Discussion

We demonstrated that alkoxy-substituted photochromic molecules can act as photo-addressable surfactant and as dispersion-stabilizing agents to enhance the yield of exfoliation in an upscalable molecule-assisted LPE-based method. The simultaneous use of UV light, promoting the *trans*-to-*cis* isomerization, as well as thermal annealing at 40 °C and mechanical forces generated by sonication, both favouring *cis*-to-*trans* isomerization, promotes the exfoliation in liquid media. The most effective exfoliation is obtained with azobenzene molecules irradiated with UV light in NMP at 40 °C, with a concentration of exfoliated graphene of 110 μg ml^−1^. This corresponds to an ∼80% increase in exfoliation yield when compared with pure NMP (63 μg ml^−1^). By depositing the hybrid film onto Au pre-patterned SiO_2_ substrates, light-responsive thin hybrid films, formed in a one-step co-deposition process, can be realized, whose conductivity can be reversibly modulated by the *trans*–*cis* photoisomerization of the azobenzenes. By combining this approach with cost-effective techniques, such as ink-jet printing, more complex responsive device designs and architectures may be realized. This paves the way to future applications such as optically controllable memory switch elements for light-assisted programming and photosensors.

## Methods

### Materials

Graphite synthetic flakes (Product No. 332461), 4-(decyloxy)azobenzene (Product No. S931950) and NMP (Product No. 270458) are sourced from Sigma-Aldrich.

### Liquid-phase exfoliation

Graphene dispersions are prepared by adding graphite powder (100 mg) to NMP (10 ml), and tip-sonication (Labsonic M, Platinum tip, diameter 2 mm, sound rating density 300 W cm^−2^) for 3 h either in dark or under UV light irradiation, using a portable laboratory ultraviolet lamp (8 W, 365 nm, 0.34 mW cm^−2^, Herolab GmbH), in glass vials (Pyrex), in the presence of azobenzene molecules (5.5 mg). The dispersions are then allowed to settle for 15 min, then decanted and centrifuged (Eppendorf, centrifuge 5804) for 1 hour at 10,000 r.p.m. From the centrifuged dispersions 70 vol% are pipetted off the top for characterization and film deposition. To quantify the concentration of flakes after centrifugation, the dispersions are passed through polytetrafluoroethylene membrane filters (pore size 100 nm). Measurements of the filtered mass are performed with a microbalance (Sartorius MSA2.75)

### Device fabrication

n^++^Si substrates with a thermally grown SiO_2_ layer (230 nm) and pre-patterned interdigitated gold source and drain electrodes (IPMS Fraunhofer) with different channel length (*L*=2.5, 5, 10 and 20 μm) and constant channel width (*W*=10 mm) are used. The substrates are cleaned before device fabrication in an ultrasonic bath (FB 15047, Fisher Scientific) of acetone and isopropanol, 15 min in each solvent, and treated 5 min (+30 min incubation) with an UV surface decontamination system (PSD-UV, Novascan) to improve wetting of the solvent. The dispersions (50 μl) are drop-cast on clean substrates and dried for 48 h in vacuum at 30 °C.

### Characterization

The electrical characterization is carried out in inert atmosphere (glovebox) with an electrometer (Keithley 2636A) interfaced with LabTracer software. For the light-induced switch, a Polychrome V monochromator (Till Photonics) is used as ultraviolet light (350 nm, 5.64 mW cm^−2^) and visible light source (450 nm, 4.83 mW cm^−2^). For the Raman experiments, graphene dispersions are drop-cast onto pre-cleaned n^++^Si substrates with a thermally grown SiO_2_ layer (300 nm) and dried for 48 h. Raman spectra are collected with a Renishaw InVia spectrometer at 457, 514.5 and 633 nm. The excitation power is kept below 1 mW to avoid the effects of local heating. The scattered light is collected with a × 100 objective. HR-TEM is done in a FEI Tecnai F20 equipped with a Schottky emitter and operated at 120 keV primary beam energy. Scanning electron microscopy images are recorded with a Quanta FEG 250 from FEI.

The thickness of the hybrid films is determined with an Alpha-Step IQ Surface Profiler from KLA Tencor. X-ray photoelectron spectroscopy analyses are carried out on a Thermo Scientific K-Alpha X-ray photoelectron spectrometer, with a basic chamber pressure ∼10^−8^ mbar and an Al anode as the X-ray source (X-ray radiation of 1,486 eV). Spot sizes of 400 μm, pass energies of 200 eV for survey scans and 50 eV for high-resolution scans are used. A volume of 150 μl of the dispersions are spin-coated on Au substrate for 1 min at 1,000 r.p.m. and substrates are annealed for 1 day at 100 °C in a oven under vacuum. Samples for X-ray diffraction are prepared by precipitating graphene and/or graphene/azobenzene by adding water:ethanol (1:1, vol:vol) into NMP. The collected precipitate is dried under vacuum for 24 h. The powder X-ray diffraction patterns are obtained using a Bruker AXS D2 Phaser (LYNXEYE detector) with Ni-filtrated Cu-Kα radiation (*λ*=1.5406 Å) with a 1-mm air-scattering slit and 0.1-mm equatorial slit. Samples are deposited on the surface of a single crystal Si wafer (cut of (911)). X-ray diffraction patterns are collected with 0.016° steps and 10 s per step increments from 8° to 80°.

## Additional information

**How to cite this article:** Döbbelin, M. *et al*. Light-enhanced liquid-phase exfoliation and current photoswitching in graphene–azobenzene composites. *Nat. Commun.* 7:11090 doi: 10.1038/ncomms11090 (2016).

## Supplementary Material

Supplementary Information Supplementary Figures 1-19, Supplementary Notes 1-8 and Supplementary References

## References

[b1] Geim A. K. & Novoselov K. S. The rise of graphene. Nat. Mater. 6, 183–191 (2007). 17330084 10.1038/nmat1849

[b2] Ferrari A. C. . Science and technology roadmap for graphene, related two-dimensional crystals, and hybrid systems. Nanoscale 7, 4598–4810 (2015). 25707682 10.1039/c4nr01600a

[b3] Bonaccorso F. . Production and processing of graphene and 2d crystals. Mater. Today 15, 564–589 (2012).

[b4] Narita A. . Synthesis of structurally well-defined and liquid-phase-processable graphene nanoribbons. Nat. Chem. 6, 126–132 (2014). 24451588 10.1038/nchem.1819

[b5] Hernandez Y. . High-yield production of graphene by liquid-phase exfoliation of graphite. Nat. Nanotechnol. 3, 563–568 (2008). 18772919 10.1038/nnano.2008.215

[b6] Chen L., Hernandez Y., Feng X. L. & Müllen K. From nanographene and graphene nanoribbons to graphene sheets: chemical synthesis. Angew. Chem. Int. Ed. 51, 7640–7654 (2012). 10.1002/anie.201201084 22777811

[b7] Palma C.-A. & Samorì P. Blueprinting macromolecular electronics. Nat. Chem. 3, 431–436 (2011). 21602856 10.1038/nchem.1043

[b8] Kim K. S. . Large-scale pattern growth of graphene films for stretchable transparent electrodes. Nature 457, 706–710 (2009). 19145232 10.1038/nature07719

[b9] Li X. S. . Large-area synthesis of high-quality and uniform graphene films on copper foils. Science 324, 1312–1314 (2009). 19423775 10.1126/science.1171245

[b10] Berger C. . Electronic confinement and coherence in patterned epitaxial graphene. Science 312, 1191–1196 (2006). 16614173 10.1126/science.1125925

[b11] Ciesielski A. & Samorì P. Graphene via sonication assisted liquid-phase exfoliation. Chem. Soc. Rev. 43, 381–398 (2014). 24002478 10.1039/c3cs60217f

[b12] Parvez K. . Electrochemically exfoliated graphene as solution-processable, highly conductive electrodes for organic electronics. ACS Nano 7, 3598–3606 (2013). 23531157 10.1021/nn400576v

[b13] Parvez K. . Exfoliation of graphite into graphene in aqueous solutions of inorganic salts. J. Am. Chem. Soc. 136, 6083–6091 (2014). 24684678 10.1021/ja5017156

[b14] León V. . Few-layer graphenes from ball-milling of graphite with melamine. Chem. Commun. 47, 10936–10938 (2011). 10.1039/c1cc14595a 21909539

[b15] Sampath S. . Direct exfoliation of graphite to graphene in aqueous media with diazaperopyrenium dications. Adv. Mater. 25, 2740–2745 (2013). 23553617 10.1002/adma.201205157

[b16] Coleman J. N. Liquid exfoliation of defect-free graphene. Acc. Chem. Res. 46, 14–22 (2013). 22433117 10.1021/ar300009f

[b17] Torrisi F. . Inkjet-printed graphene electronics. ACS Nano 6, 2992–3006 (2012). 22449258 10.1021/nn2044609

[b18] Marago O. M. . Brownian motion of graphene. ACS Nano 4, 7515–7523 (2010). 21133432 10.1021/nn1018126

[b19] Ciesielski A. . Liquid-phase exfoliation of graphene using intercalating compounds: a supramolecular approach. Angew. Chem. Int. Ed. 53, 10355–10361 (2014). 10.1002/anie.201402696 25044532

[b20] Haar S. . A supramolecular strategy to leverage the liquid-phase exfoliation of graphene in the presence of surfactants: unraveling the role of the length of fatty acids. Small 11, 1691–1702 (2014). 25504589 10.1002/smll.201402745

[b21] Luo W. . A high energy density azobenzene/graphene hybrid: a nano-templated platform for solar thermal storage. J. Mater. Chem. A 3, 11787–11795 (2015).

[b22] Peimyoo N. . Photocontrolled molecular structural transition and doping in graphene. ACS Nano 6, 8878–8886 (2012). 22966836 10.1021/nn302876w

[b23] Seo S., Min M., Lee S. M. & Lee H. Photo-switchable molecular monolayer anchored between highly transparent and flexible graphene electrodes. Nat. Commun. 4, 1920 (2013). 23715279 10.1038/ncomms2937

[b24] Kim M., Safron N. S., Huang C. H., Arnold M. S. & Gopalan P. Light-driven reversible modulation of doping in graphene. Nano Lett. 12, 182–187 (2012). 22149166 10.1021/nl2032734

[b25] Margapoti E. . Emergence of photoswitchable states in a graphene–azobenzene–Au platform. Nano Lett. 14, 6823–6827 (2014). 25414977 10.1021/nl503681z

[b26] Mativetsky J. M. . Azobenzenes as light-controlled molecular electronic switches in nanoscale metal-molecule-metal junctions. J. Am. Chem. Soc. 130, 9192–9193 (2008). 18576645 10.1021/ja8018093

[b27] Feng Y. Y., Zhang X. Q., Ding X. S. & Feng W. A light-driven reversible conductance switch based on a few-walled carbon nanotube/azobenzene hybrid linked by a flexible spacer. Carbon 48, 3091–3096 (2010).

[b28] Rabe J. P. & Buchholz S. Commensurability and mobility in 2-dimensional molecular-patterns on graphite. Science 253, 424–427 (1991). 17746397 10.1126/science.253.5018.424

[b29] Schlierf A. . Nanoscale insight into the exfoliation mechanism of graphene with organic dyes: effect of charge, dipole and molecular structure. Nanoscale 5, 4205–4216 (2013). 23467481 10.1039/c3nr00258f

[b30] Yang H. F. . Dielectric nanosheets made by liquid-phase exfoliation in water and their use in graphene-based electronics. 2D Mater. 1, 011012 (2014).

[b31] Griffiths J. Photochemistry of azobenzene and its derivatives. Chem. Soc. Rev. 1, 481–493 (1972).

[b32] Tamai N. & Miyasaka H. Ultrafast dynamics of photochromic systems. Chem. Rev. 100, 1875–1890 (2000). 11777424 10.1021/cr9800816

[b33] Bandara H. M. D. & Burdette S. C. Photoisomerization in different classes of azobenzene. Chem. Soc. Rev. 41, 1809–1825 (2012). 22008710 10.1039/c1cs15179g

[b34] Joseph J. M., Destaillats H., Hung H. M. & Hoffmann M. R. The sonochemical degradation of azobenzene and related azo dyes: rate enhancements via Fenton's reactions. J. Phys. Chem. A 104, 301–307 (2000).

[b35] Turansky R., Konopka M., Doltsinis N. L., Stich I. & Marx D. Switching of functionalized azobenzene suspended between gold tips by mechanochemical, photochemical, and opto-mechanical means. Phys. Chem. Chem. Phys. 12, 13922–13932 (2010). 20844786 10.1039/c0cp00588f

[b36] Surampudi S. K., Patel H. R., Nagarjuna G. & Venkataraman D. Mechano-isomerization of azobenzene. Chem. Commun. 49, 7519–7521 (2013). 10.1039/c3cc43797c 23864053

[b37] Shin K. H. & Shin E. J. Photoresponsive azobenzene-modified gold nanoparticle. Bull. Korean Chem. Soc. 29, 1259–1262 (2008).

[b38] Ferrari A. C. . Raman spectrum of graphene and graphene layers. Phys. Rev. Lett. 97, 187401–187405 (2006). 17155573 10.1103/PhysRevLett.97.187401

[b39] Ferrari A. C. & Robertson J. Raman spectroscopy of amorphous, nanostructured, diamond-like carbon, and nanodiamond. Philos. Trans. R. Soc. Lond. Ser. A 362, 2477–2512 (2004). 10.1098/rsta.2004.1452 15482988

[b40] Ferrari A. C. & Basko D. M. Raman spectroscopy as a versatile tool for studying the properties of graphene. Nat. Nanotechnol. 8, 235–246 (2013). 23552117 10.1038/nnano.2013.46

[b41] Tuinstra F. & Koenig J. L. Raman spectrum of graphite. J. Chem. Phys. 53, 1126–1130 (1970).

[b42] Tan P. H. . The shear mode of multilayer graphene. Nat. Mater. 11, 294–300 (2012). 22306771 10.1038/nmat3245

[b43] Lui C. H. . Observation of layer-breathing mode vibrations in few-layer graphene through combination Raman scattering. Nano Lett. 12, 5539–5544 (2012). 22963681 10.1021/nl302450s

[b44] Ferrari A. C. & Robertson J. Resonant Raman spectroscopy of disordered, amorphous, and diamondlike carbon. Phys. Rev. B 64, 075414–075426 (2001).

[b45] Ferrari A. C. & Robertson J. Interpretation of Raman spectra of disordered and amorphous carbon. Phys. Rev. B 61, 14095–14107 (2000).

[b46] Thomsen C. & Reich S. Double resonant Raman scattering in graphite. Phys. Rev. Lett. 85, 5214–5217 (2000). 11102224 10.1103/PhysRevLett.85.5214

[b47] Piscanec S., Lazzeri M., Mauri F., Ferrari A. C. & Robertson J. Kohn anomalies and electron-phonon interactions in graphite. Phys. Rev. Lett. 93, 185503 (2004). 15525177 10.1103/PhysRevLett.93.185503

[b48] Zheng Y. B. . Surface-enhanced Raman spectroscopy to probe reversibly photoswitchable azobenzene in controlled nanoscale environments. Nano Lett. 11, 3447–3452 (2011). 21749070 10.1021/nl2019195

[b49] Chunder A., Pal T., Khondaker S. I. & Zhai L. Reduced graphene oxide/copper phthalocyanine composite and its optoelectrical properties. J. Phys. Chem. C 114, 15129–15135 (2010).

[b50] Abraham M. J. . GROMACS: high performance molecular simulations through multi-level parallelism from laptops to supercomputers. SoftwareX 1, 19–25 (2015).

[b51] Schafer L. V., Muller E. M., Gaub H. E. & Grubmuller H. Elastic properties of photoswitchable azobenzene polymers from molecular dynamics simulations. Angew. Chem. Int. Ed. 46, 2232–2237 (2007). 10.1002/anie.200604595 17300120

[b52] Pipolo S. . First-principle-based MD description of azobenzene molecular rods. Theor. Chem. Acc. 131, 1274–1287 (2012).

[b53] Ridley J. & Zerner M. An intermediate neglect of differential overlap technique for spectroscopy: pyrrole and the azines. Theor. Chem. Acc. 32, 111–134 (1973).

[b54] Kondov I., Cizek M., Benesch C., Wang H. B. & Thoss M. Quantum dynamics of photoinduced electron-transfer reactions in dye-semiconductor systems: first-principles description and application to coumarin 343-TiO_2_. J. Phys. Chem. C 111, 11970–11981 (2007).

