# Supplementary material for: Light-enhanced liquid-phase exfoliation and current photoswitching in graphene–azobenzene composites
Source: Nat Commun. 2016 Apr 7;7:11090. doi: 10.1038/ncomms11090 (PMC4829665; doi:10.1038/ncomms11090)
Supplement: Supplementary Information — Supplementary Figures 1-19, Supplementary Notes 1-8 and Supplementary References [file ncomms11090-s1.pdf]

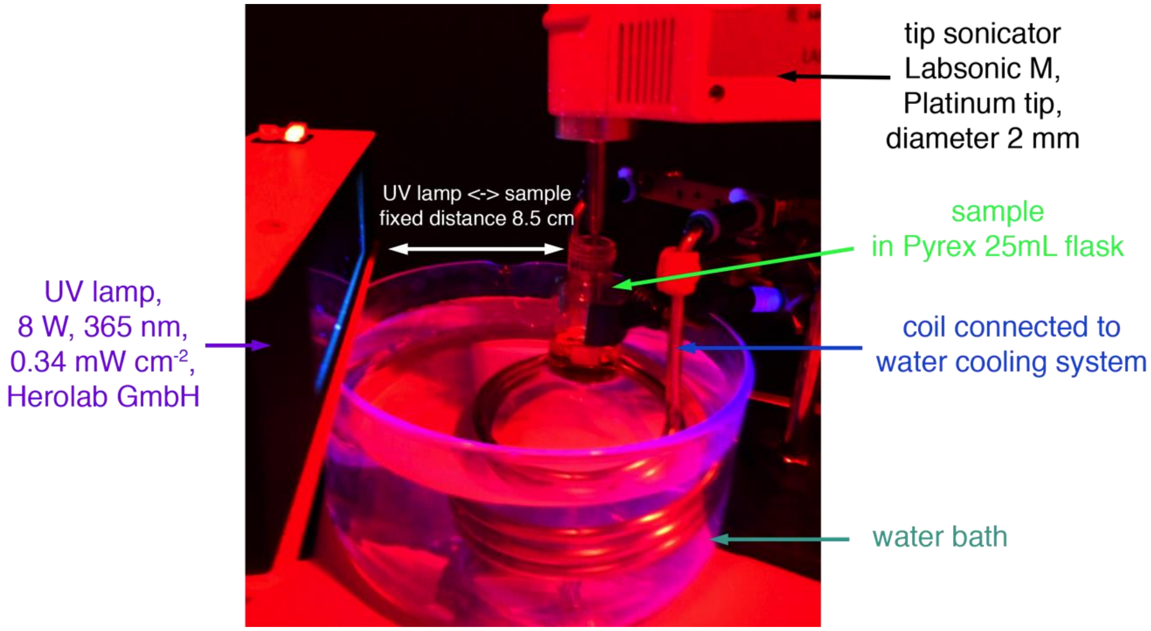

Supplementary Figure 1 | Photograph of experimental set-up

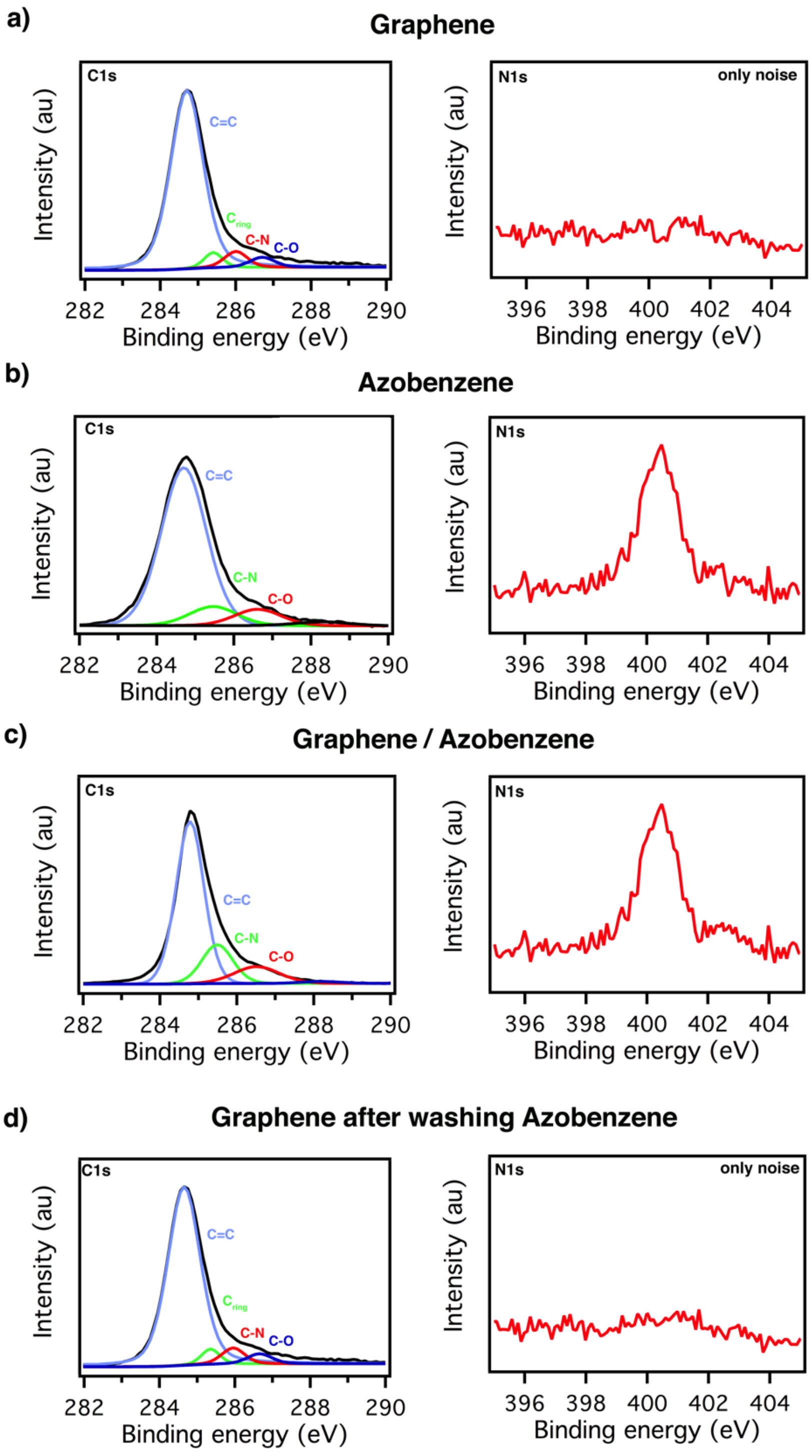

Supplementary Figure 2 | High-resolution C1s and N1s spectra of (a) flakes exfoliated in NMP, (b) C1s and N1s spectra of 4-(decyloxy)-azobenzene flakes, (c) exfoliated in NMP at 40 °C in the presence of 4-(decyloxy)-azobenzene molecules, and (d) after the washing step. Experimental data are shown as black line, and the individual deconvolved components as coloured lines.

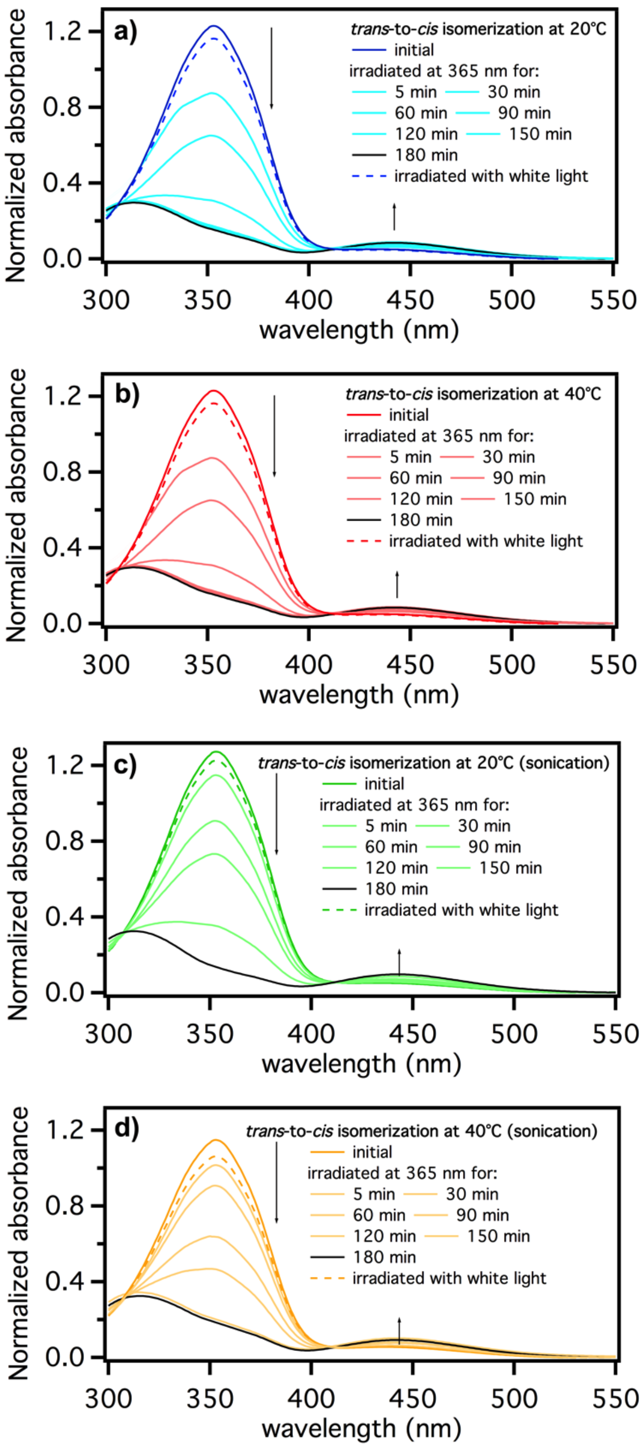

Supplementary Figure 3 | UV-vis spectra of *trans*-to-*cis* isomerization of 4-(decyloxy)azobenzene in NMP showing the disappearance of the 353 nm band and the increase of the 442 nm band upon irradiation with UV-light (λ = 365 nm) for 3h under different experimental conditions: a) 20 °C, b) 40 °C, c) 20 °C / sonicated and d) 40 °C / sonicated.

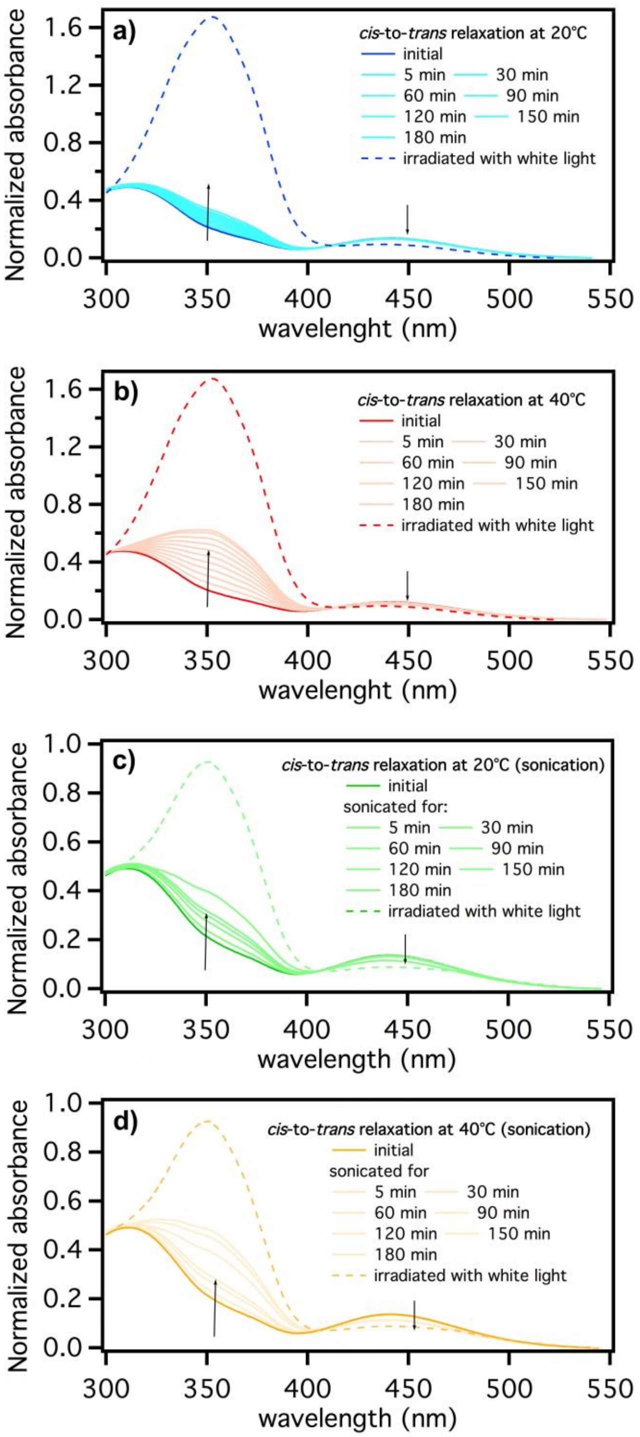

Supplementary Figure 4 | UV-vis spectra the *cis*-to-*trans* relaxation of *cis*-4-(decyloxy)azobenzene in NMP at (a) 20 °C and (b) 40 °C, and *cis*-to-*trans* mechano-relaxation (exposed to ultrasounds) at (c) 20 °C and (d) 40 °C showing the disappearance of the 442 nm band and the increase of the 353 nm band as a function of time. All samples are kept in dark to avoid light affecting the isomerisation of 4-(decyloxy)azobenzene.

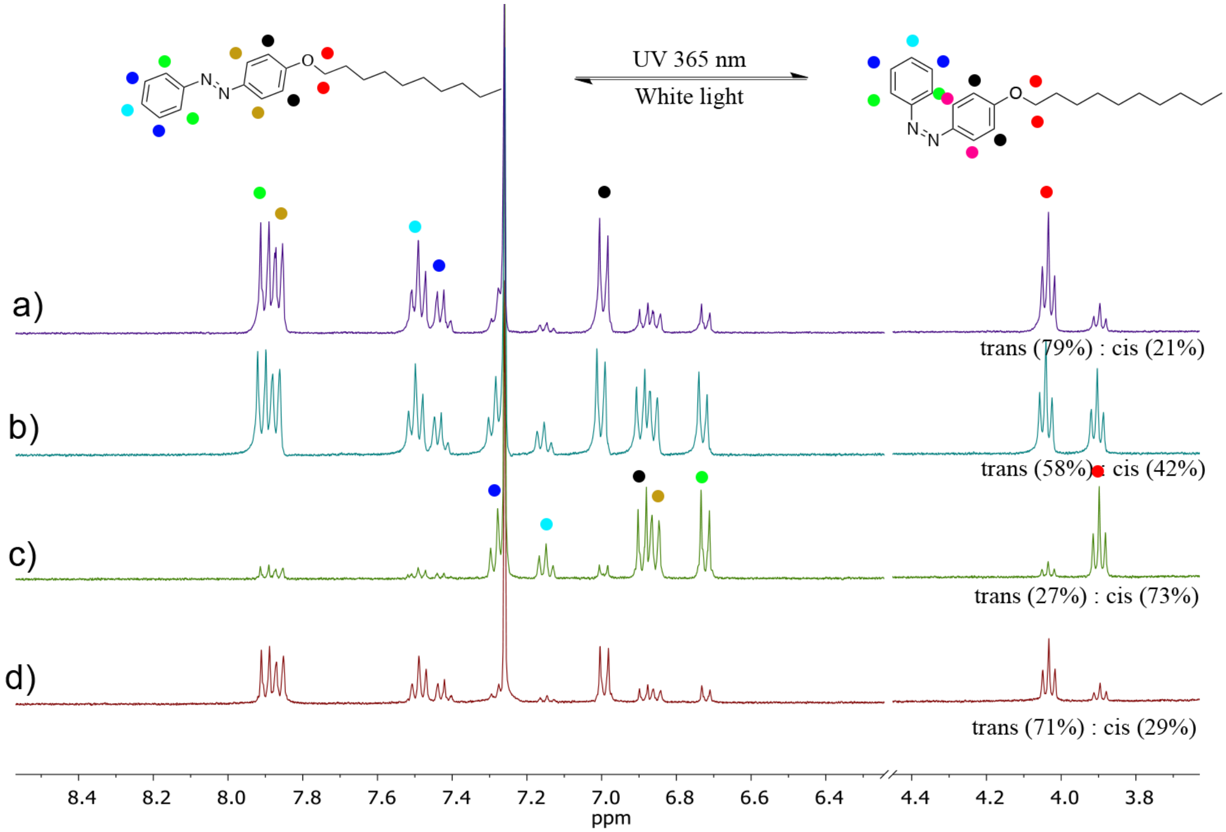

Supplementary Figure 5 | ^{1}H-NMR spectra of 4-(decyloxy)azobenzene in NMP:$CDCl_3$ (1:30 *vol:vol*, 1.6 mM): a) initial spectrum consisting of 79 and 21% of *trans* and *cis*, respectively; b) spectrum of the solution irradiated for 1 h at 365 nm consisting of 58 and 42% of *trans* and *cis*, respectively; c) spectrum of the solution irradiated for 2.5 h at 365 nm consisting of 27 and 73% of *trans* and *cis*, respectively; d) spectrum of the solution (c) irradiated with white light consisting of 71 and 29% of *trans* and *cis*, respectively.

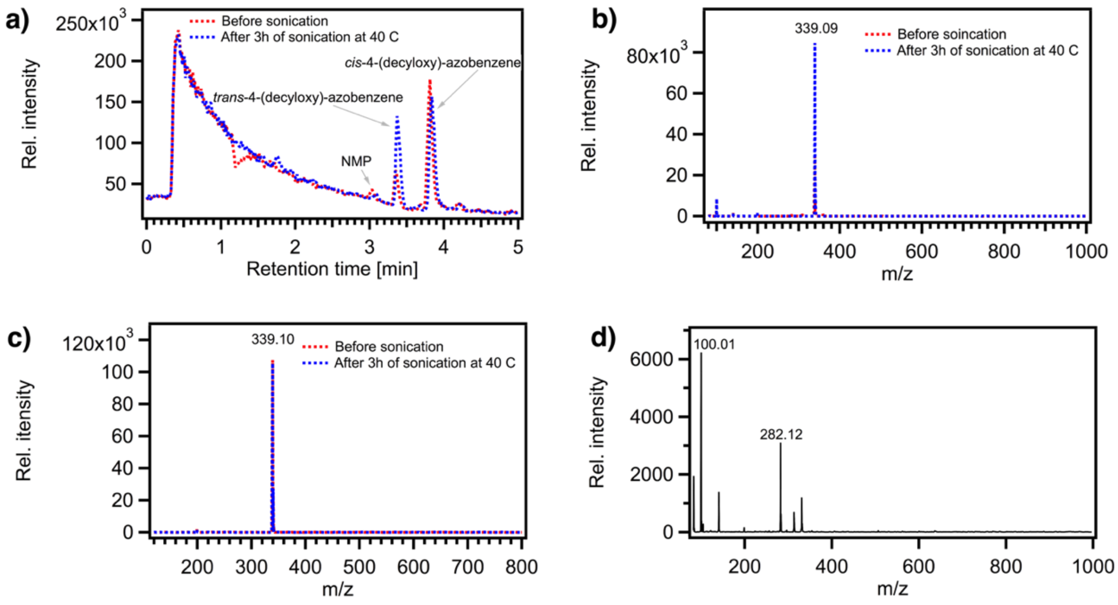

Supplementary Figure 6 | a) HPLC chromatogram showing the distribution of species obtained from the of the 4-(decyloxy)-azobenzene solution before and after sonication for 3h at 40 °C; m/z spectra of peaks at the retention time R_t = 3.37 min, 3.84 min and 3.1 min are shown in supplementary figures (b), (c) and (d), respectively.

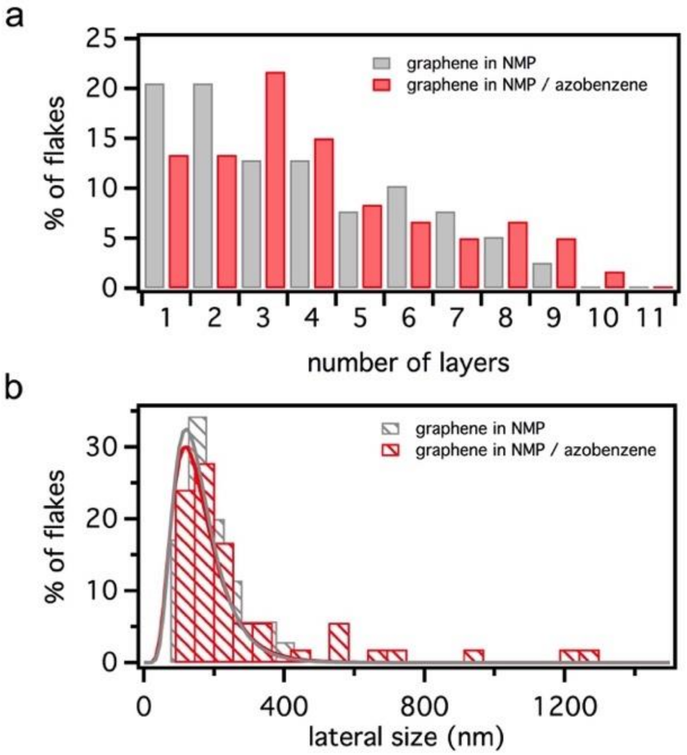

Supplementary Figure 7 | HR-TEM statistics. (a) N distribution for LPE in (grey) NMP and (red) NMP/azobenzene. (b) Lateral flake size distribution, fitted with a lognormal distribution.

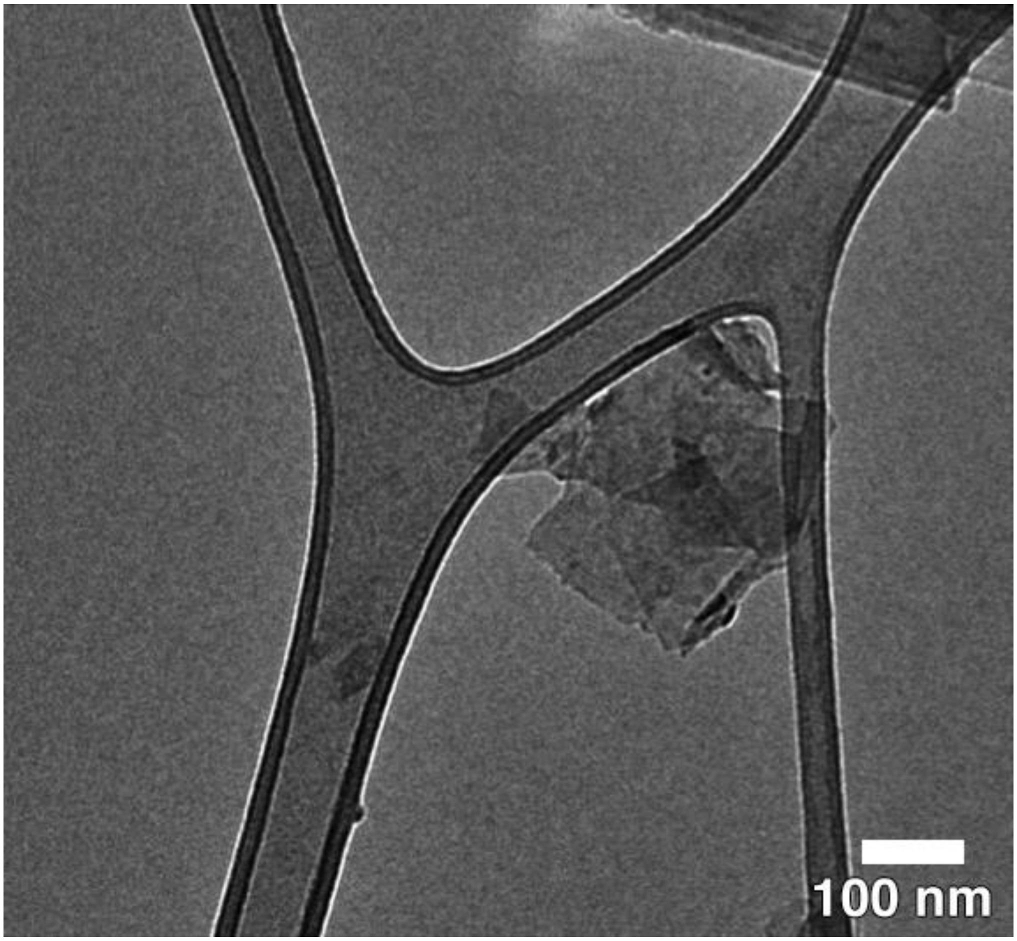

Supplementary Figure 8 | Low magnification TEM image of an azobenzene-exfoliated flake.

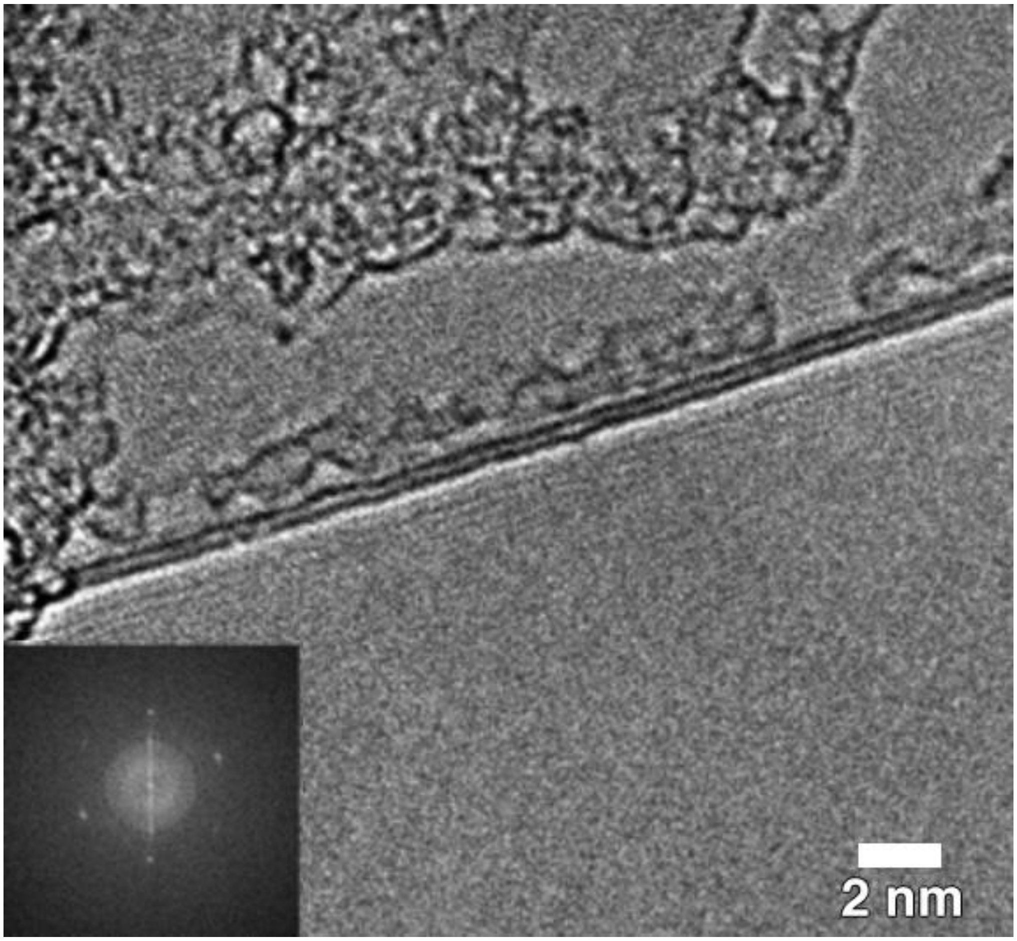

Supplementary Figure 9 | High Resolution image of azobenzene-exfoliated double-layer. The corresponding FFT is shown in the inset.

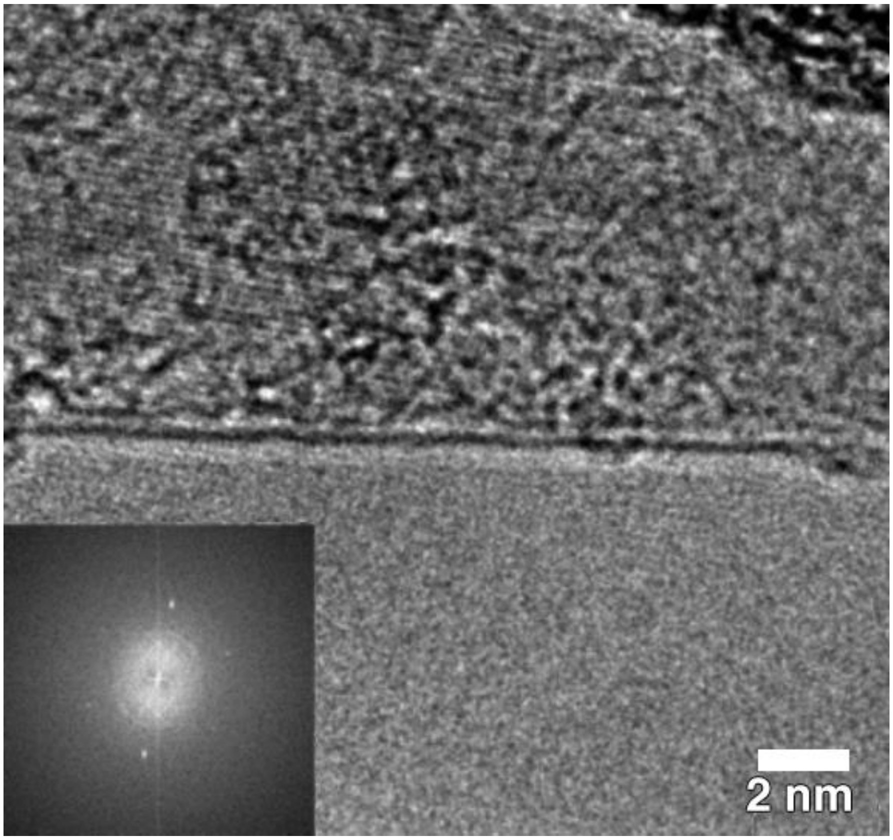

Supplementary Figure 10 | High Resolution image of an azobenzene-exfoliated SLG. The corresponding FFT is shown in the inset.

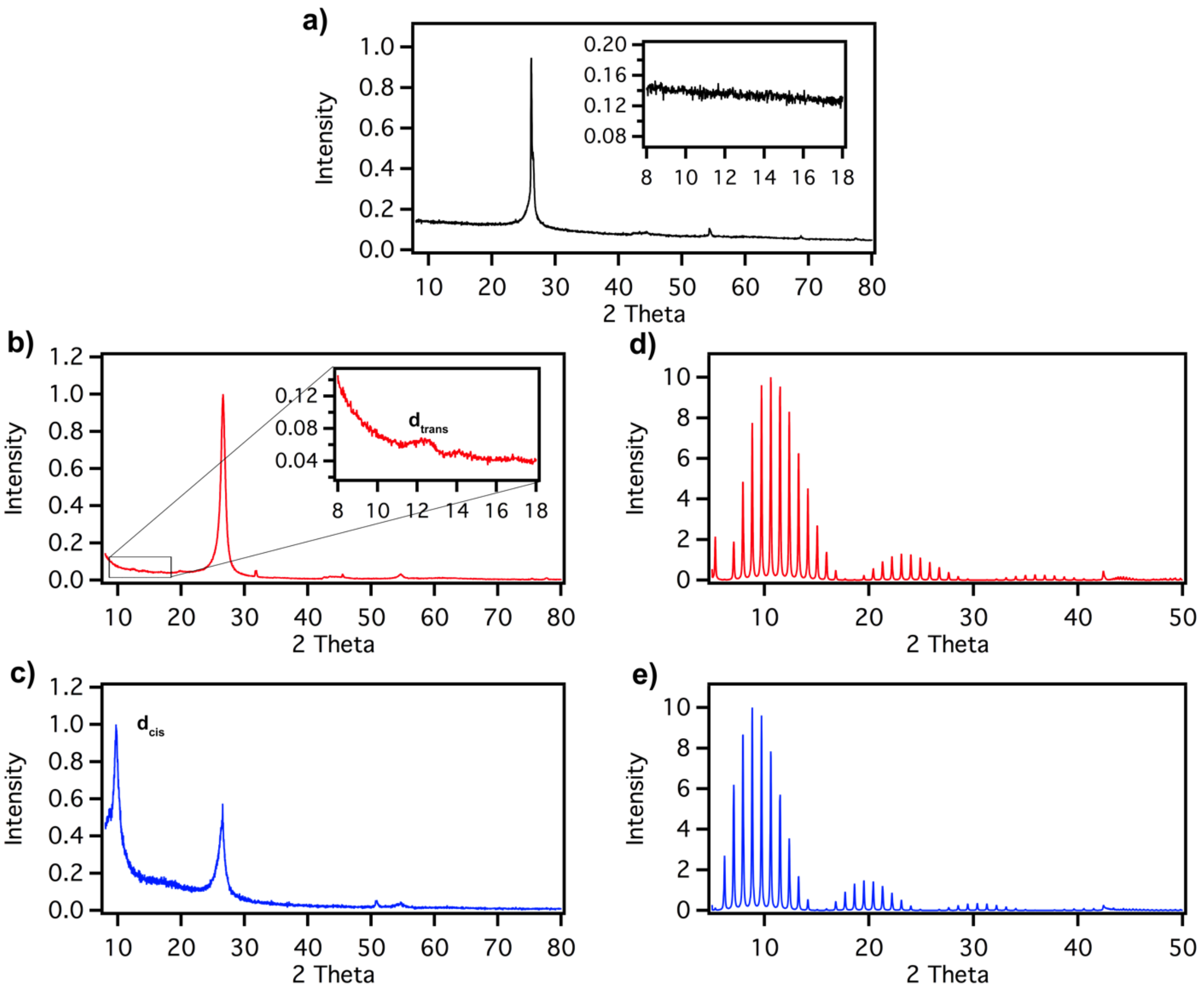

Supplementary Figure 11 | XRD patterns of (a) reference sample, (b) hybrid material composed of graphene/*trans*-4-(decyloxy)azobenzene, (c) and graphene/*cis*-4-(decyloxy)azobenzene. Simulated XRD patterns of calculated structures of (d) *trans*- and (e) *cis*-4-(decyloxy)azobenzene intercalating graphene sheets.

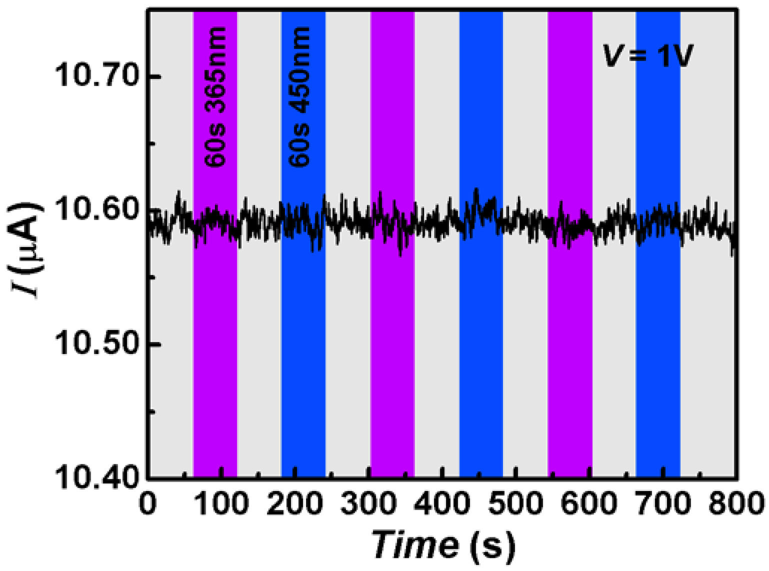

Supplementary Figure 12 | Current response of a reference film for a static bias and dynamic alternating UV and visible irradiation cycles (the channel length between Au electrodes is 10 μm).

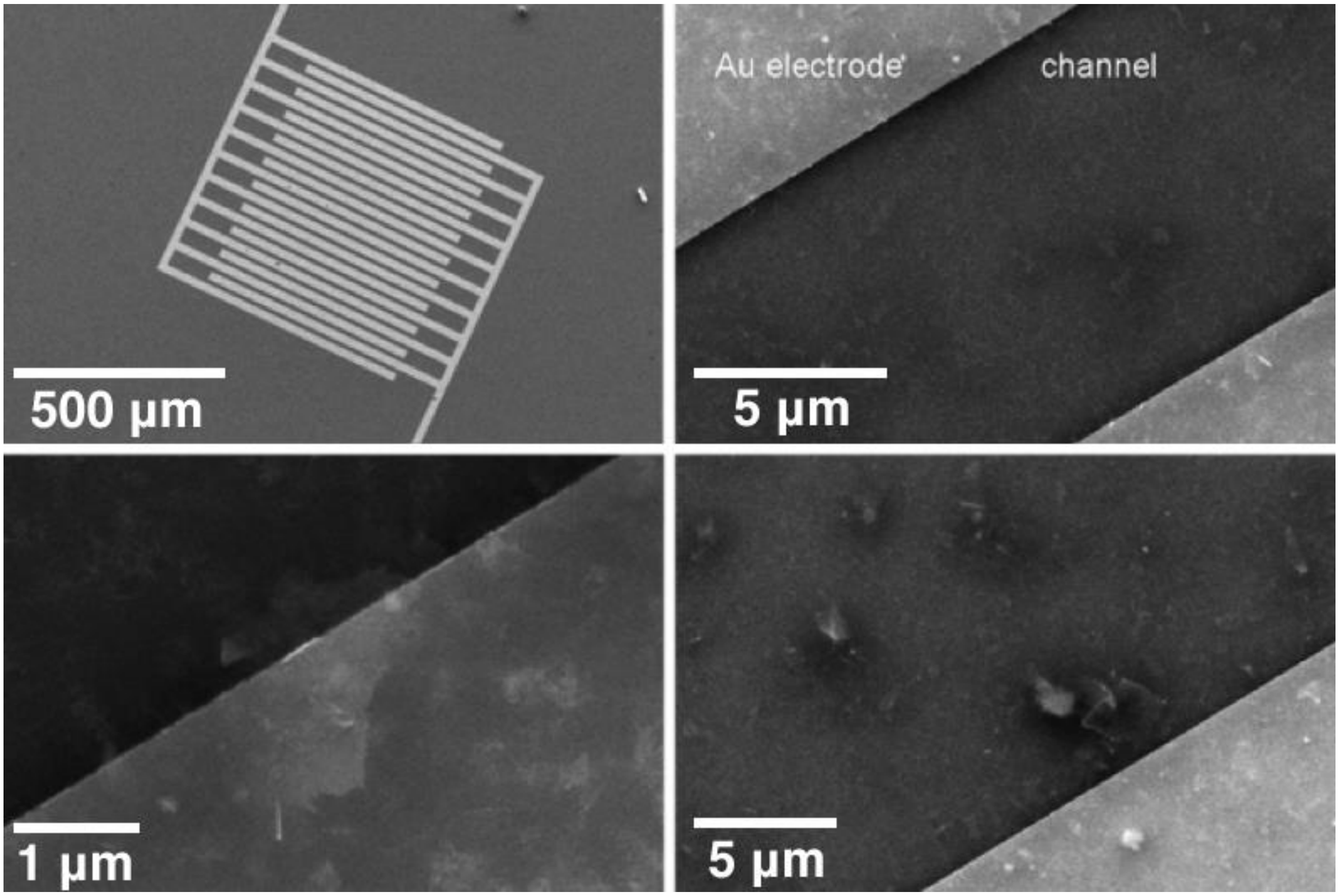

Supplementary Figure 13 | SEM images of exfoliated flakes on substrates (channel length = 10 µm) with interdigitated Au electrodes.

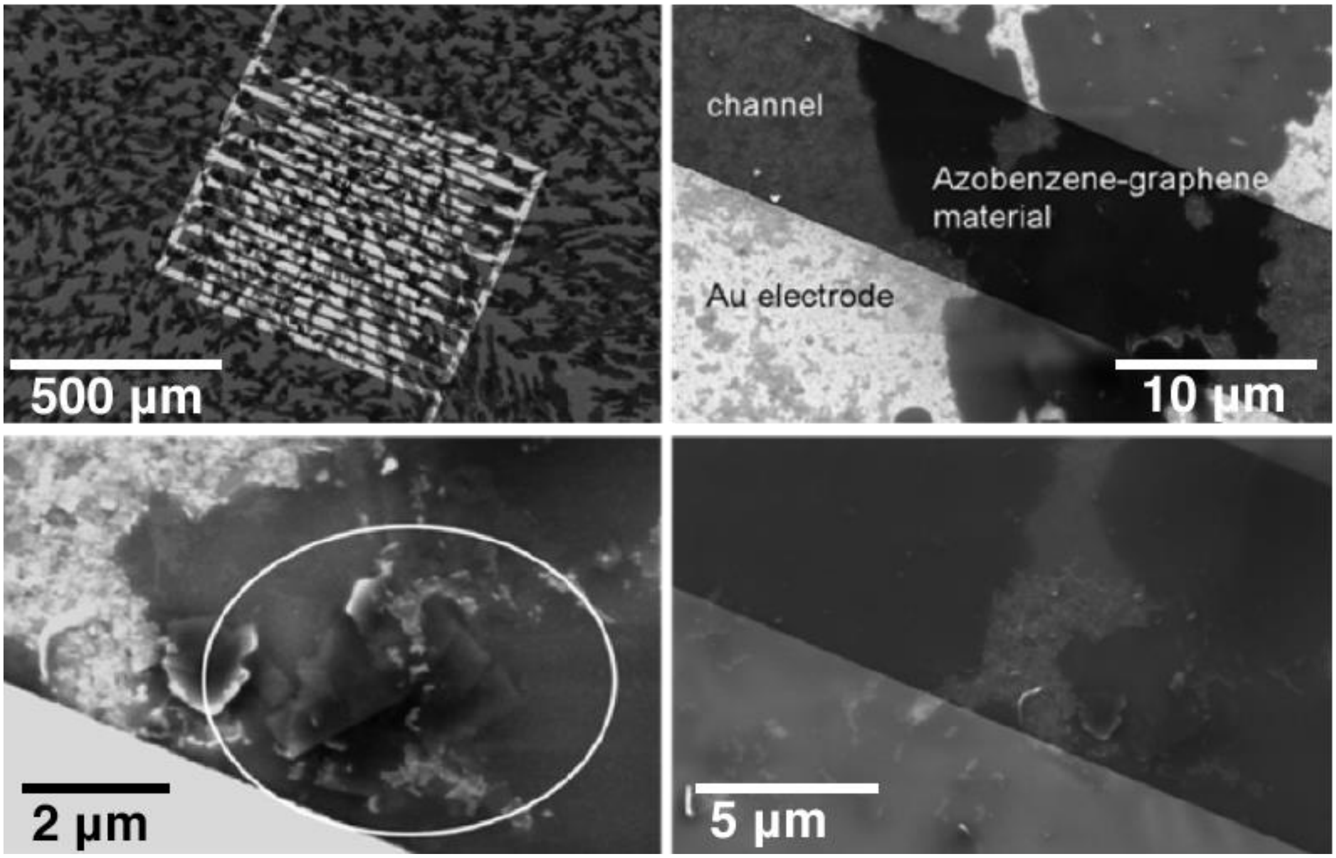

Supplementary Figure 14 | SEM images of graphene/4-(decyloxy)azobenzene on substrates with interdigitated Au electrodes. The substrates are well covered and the electrodes are bridged. The flakes (white circle) are dispersed in the 4-(decyloxy)azobenzene matrix.

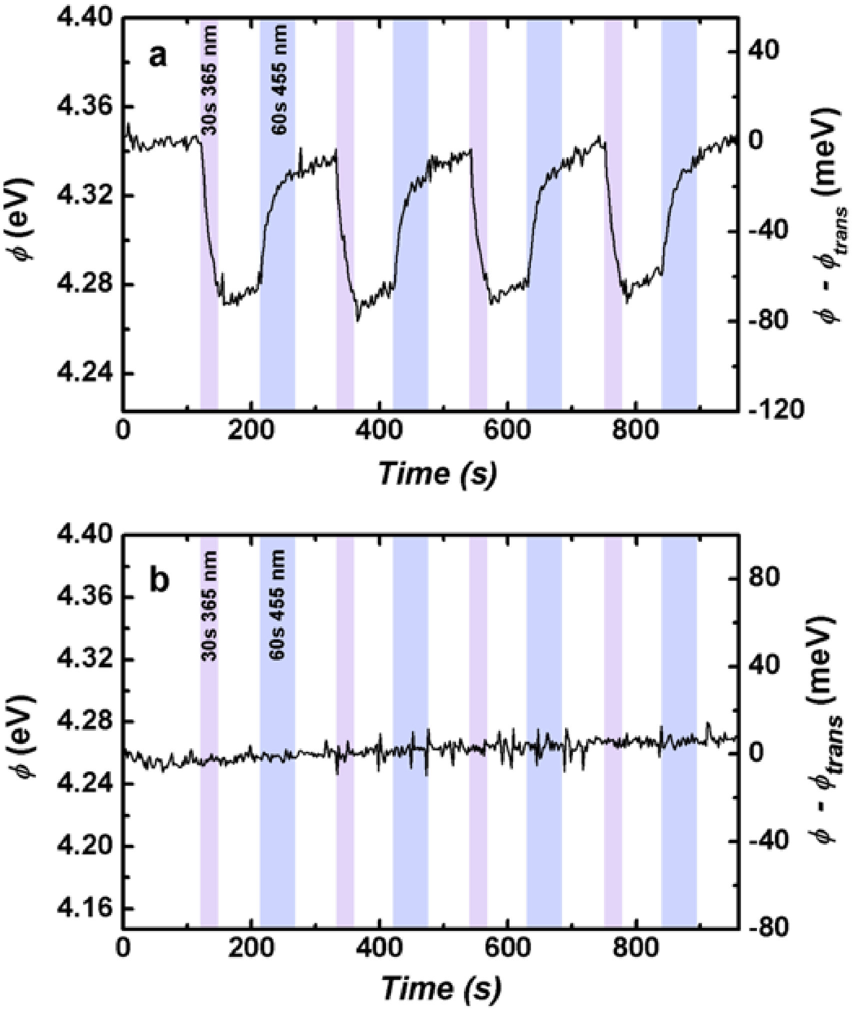

Supplementary Figure 15 | Macroscopic Kelvin Probe measurements under repeated light irradiation cycles of a (a) graphene-azobenzene hybrid and (b) reference film.

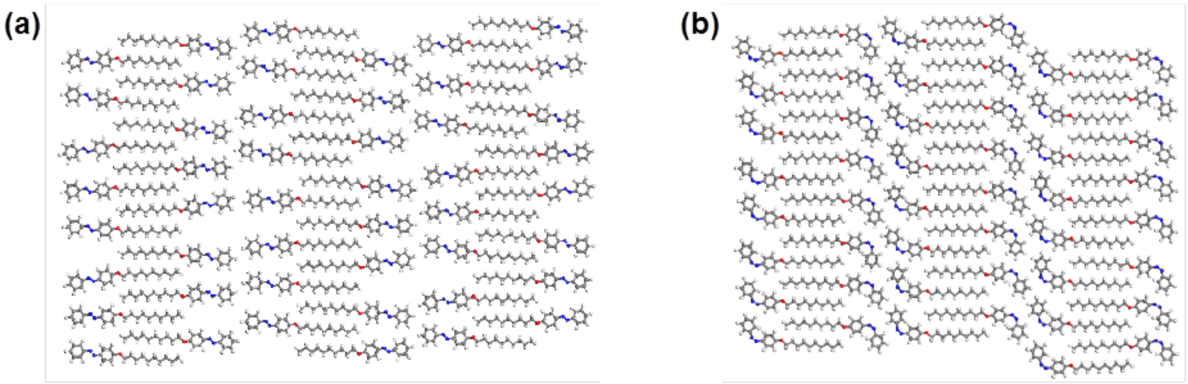

Supplementary Figure 16 | Ordered self-assembled monolayers of (a) *trans* and (b) *cis* azobenzenes. The graphene surface is not shown for clarity.

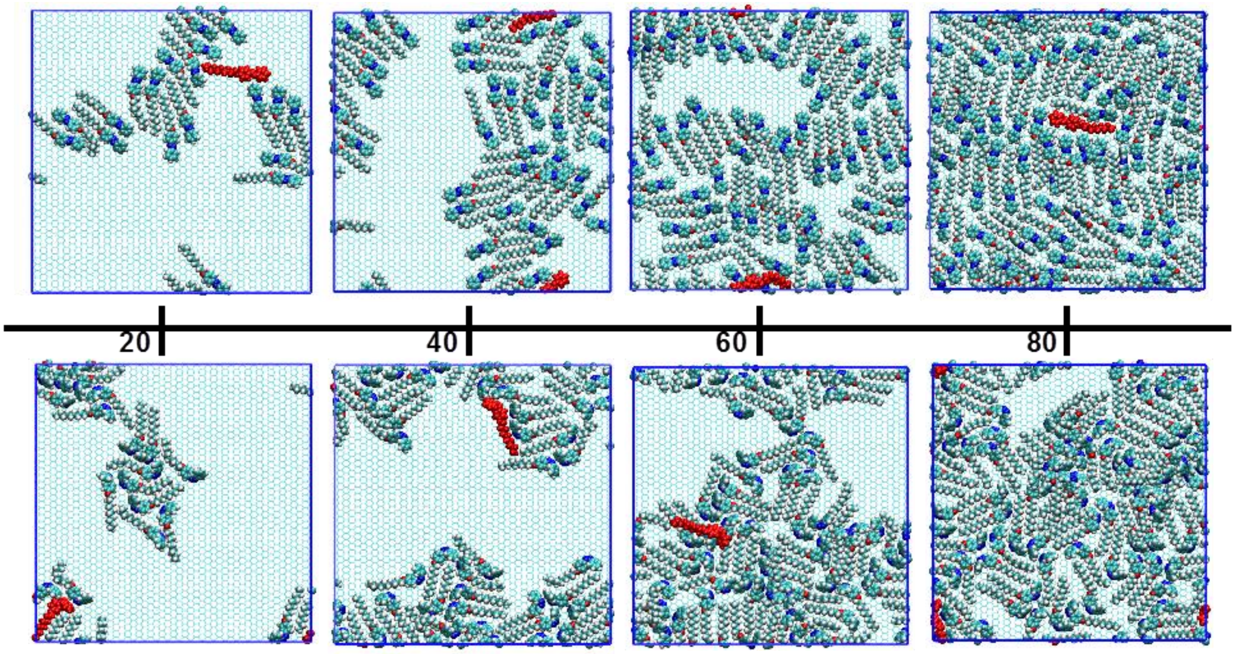

Supplementary Figure 17 | Snapshots of the deposition process with 20, 40, 60 and 80 molecules of *trans* (above) and *cis* (below) azobenzene. (Red) last molecules added for each snapshot.

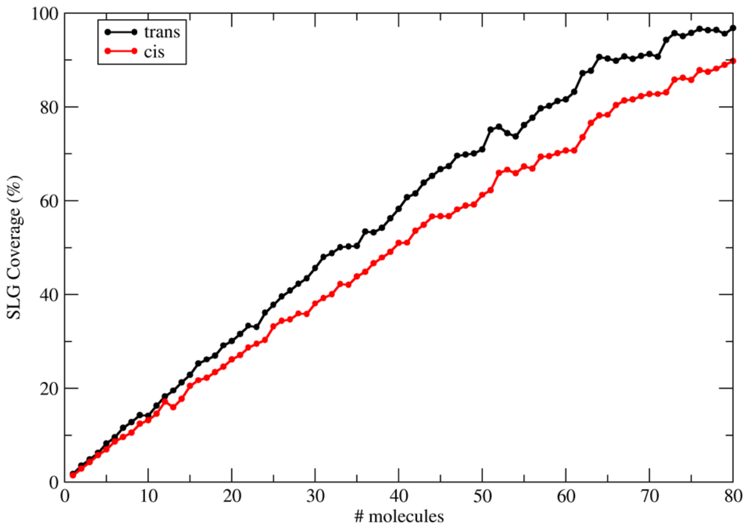

Supplementary Figure 18 | Coverage of graphene as a function of the number of (red) *cis* and (black) *trans* azobenzene molecules.

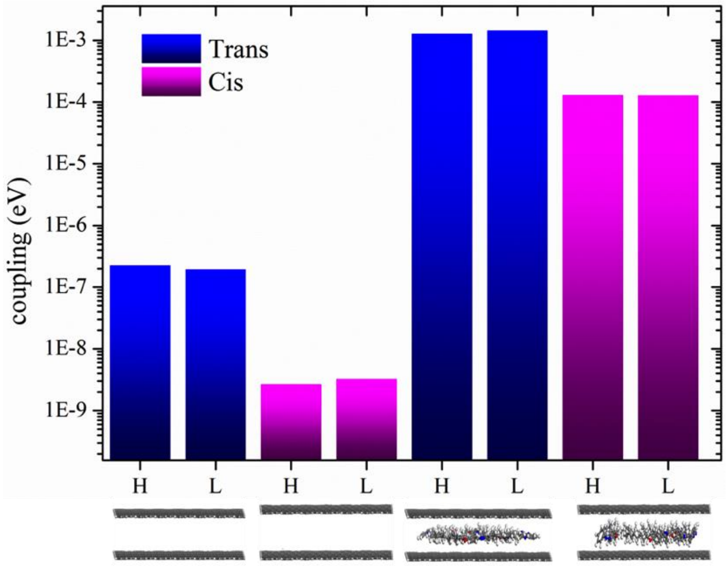

Supplementary Figure 19 | (left) Coupling calculated for the dimer (not sandwiched layer) with interlayer distances of 0.73 nm (*trans* conformation) and 0.85 nm (*cis* conformation), and (right) *in presence* of the sandwiched azobenzenes for the *trans* (blue bars) and *cis* (magenta bars) isomers. H and L refer to electronic transfer integrals for the HOMO (hole) or LUMO (electron) of graphene sheets.

Supplementary Note 1

We calculate the number of molecules needed to form densely packed azobenzene monolayers on SLG.[1] We use 1- 10% of 4-(decyloxy)azobenzene molecules to cover the entire SLG surface. We first exfoliate graphite with different weight percentages of 4-(decyloxy)azobenzene: 1 – 5 %. Y_W does not increase up to 4%. Above 5 % Y_W is similar to that measured at 4%. However, above 5% we detect a decrease in conductivity, due to the increased amount of azobenzene insulating phase. Thus, in order to keep good electrical performance of the devices based on the hybrid material we focus our the LPE process assisted by 4% of azobenzene molecules. All dispersions are prepared by placing graphite powder (100 mg) in NMP (10 mL), followed by tip-sonication (Labsonic M, Platinum tip, diameter 2 mm) for 3 hours either in dark or under UV light irradiation using a portable laboratory UV lamp (8 W, 365 nm, 0.34 mW cm^{-2}, Herolab GmbH), at 20 and 40 °C in glass vials (25 mL, Pyrex), in the presence of azobenzene molecules (5.5 mg). Supplementary Figure 1 shows the experimental *set-up*.

Supplementary Note 2

XPS analyses are carried out on a Thermo Scientific K-Alpha spectrometer with a base pressure ~10^{-8} mbar and an Al anode as the X-ray source (X-ray radiation of 1486 eV). Spot sizes of 400 μm are used and pass energies of 200.00 eV for survey scans and 50.00 eV for high-resolution scans. 150 μL is spin coated on Au for 1 minute at 1000 rpm followed by annealing for 1 day at 100 °C in a vacuum oven.

We use XPS to analyse graphene/4-(decyloxy)-azobenzene dispersions in NMP before and after washing out the azobenzene molecules. The high-resolution C1s XPS spectrum of graphene shows a sharp peak at 284.7 eV, corresponding to C=C bonds in a conjugated honeycomb lattice (Supplementary Fig. 2a).[2] The peak at 285.7 eV can be attributed to C_{ring}. The peaks at and 286 eV and 286.6 can be assigned to C-N and C-O bonds, respectively,[2] and are attributed to remaining solvent molecules, i.e. NMP. The high-resolution N1s XPS spectrum of the reference sample does not show any nitrogen signal (Supplementary Fig. 2a).

The C1s XPS spectrum of the 4-(decyloxy)-azobenzene molecules (Supplementary Fig. 2b) shows a main peak at 284.6 eV (C-C) and two smaller peaks at 285.4 eV and 286.6 eV. These are attributed to C-N and C-O bonds, respectively.[3] The peak at ~400.4 eV in N1s spectrum corresponds to C-N (Supplementary Fig. 2b).

The C1s spectrum of the graphene/4-(decyloxy)-azobenzene hybrid film (Supplementary Fig. 2a) has three peaks, including a sharp one at 284.7 eV, assigned C-C bonds, as in the case of the graphene spectrum (Supplementary Fig. 2a).[4] A peak at 285.4 is attributed to the presence of both C-N and C_{ring}, whereas that at 286.6 is assigned to C-O. Similarly to the films of 4-(decyloxy)-azobenzene molecules, the N1s spectrum of the graphene/4-(decyloxy)-azobenzene hybrid shows a peak at ~400.4 eV.

After the washing the graphene/4-(decyloxy)-azobenzene hybrid, the intensities of all C-N and C-O peaks significantly decrease (Supplementary Fig. 2d), until reaching the same level as in the reference sample, indicating the removal of azobenzene, as further confirmed by the absence of C-N peak in N1s spectrum.

Supplementary Note 3

A solution of 4-(decyloxy)-azobenzene is prepared by dissolving the molecules in NMP to 10^{-3} M. These are then irradiated with UV light at for 30 min and sonicated at 40 °C for 3h in dark. The solution is then diluted with CH_3CN at 10^{-5} M. The High-Pressure Liquid Chromatography (HPLC) experiment is performed using Thermo scientific Accela LC system with an hypersil gold column. During assays, 10 μL of each sample is injected into the analytic column. HPLC trace of the compound, before sonication, shows two peaks at the retention time R_t = 3.37 min and 3.84 min (red dots in Supplementary Fig. 6a). The peak at 3.37 min is assigned to *trans*-4-(decyloxy)-azobenzene (see red dots in Supplementary Fig. 6b for m/z) and the peak at 3.84 is assigned to the *cis* conformer (see red dots in Supplementary Fig. 6c for m/z). Such assignment is consistent with our NMR and spectroscopic observations, where the photostationary state (PSS) of 4-(decyloxy)-azobenzene after irradiation with UV light is composed by~20% *trans* and 80% *cis* isomers (see Supplementary Figure 5). The sonication of the solution for 3h at 40 °C results in identical peak distribution (R_t = 3.34 min and R_t = 3.81 min) with a visible change of peaks intensities (blue dots in Supplementary Fig. 6a), which can be explained by the fact the upon sonication at 40 °C, *cis*-4-(decyloxy)-azobenzene isomerize to the *trans* form. The ratio between the *trans* and cis *isomer* (ca 1:1) is in a good agreement with NMR and spectroscopic data (see Supplementary Figure 5). The peak at R_t = 3.1 min (red and blue dots in Supplementary Fig. 6a) is assigned to NMP molecules, as evidenced by its mass-to-charge ratio (m/z) in Supplementary Fig. 6d. These results demonstrate that the compound remains intact during sonication, which reflects its stability in NMP even upon sonication at 40 °C.

Supplementary Note 4

HR-TEM is carried out to determine the thickness as well as the lateral dimensions of the exfoliated flakes. TEM samples are prepared by drop casting on Quantifoil holey carbon copper grids, followed by solvent evaporation at 150 °C for 10 min. TEM micrographs reveal folded SLG, with lateral sizes below 1 μm, as typically observed for LPE.[2, 5-7] Supplementary Figure 7 shows a statistical distribution of N and lateral flake sizes for samples prepared in the absence and the presence of 4-(decyloxy)azobenzene molecules. Supplementary Figure 8 shows a low magnification image of azobenzene-exfoliated flakes deposited on a holey carbon grid. The flakes have a lateral dimension of hundreds of nanometres.

Supplementary Figure 9 is a high-resolution image of a folded flake comprising two SLGs, as highlighted by the 002 fringes along the border. Despite some contaminants, residuals of the deposition process, the crystalline structure in the internal part of the flake is visible, highlighted also by the Fast Fourier Transform pattern in the inset, showing the expected hexagonal pattern. Similarly, Supplementary Figure 10 depicts a HR-TEM of a folded SLG, with the corresponding FFT pattern in the inset.

Supplementary Note 5

Powder X-ray diffraction patterns are further recorded to characterize the structure of the hybrid films and compare them to control samples. The reference samples display a sharp peak at 26.7^o, corresponding to an interlayer spacing of ~0.33 nm (see Supplementary Fig. 11a). The XRD pattern of graphene/*trans*-azobenzene films (Supplementary Fig. 11b) shows a peak at 2θ = 12.3° ($d_{spacing}$ ~0.72 nm), evidencing that the interlayer spacing depends on the 4-(decyloxy)azobenzene conformation. The XRD pattern of graphene/*cis*-4-(decyloxy)azobenzene films (Supplementary Fig. 11c) shows a new peak at 2θ = 9.9° (see inset in Supplementary Fig. 11b), corresponding to an interlayer spacing of ~0.89 nm. The broad peak at 26° originates from the partial restacking of non-intercalated graphene sheets. Supplementary Fig. 11d,f show simulated (using modelled sandwich structures) XRD patterns for graphene/*trans*-4-(decyloxy)azobenzene and graphene/*cis*-4-(decyloxy)azobenzene, respectively.

Supplementary Note 6

Kelvin Probe can be used to measure the work function (Φ) of organic and inorganic layers with high resolution[8, 9] (1-3 meV) over a long time period (up to 60 min).

Samples are prepared by drop-casting on Au/Mica substrates, followed by drying for 48 h at 30 °C in vacuum. Measurements are performed in ambient conditions using a 2 mm gold tip (Ambient Kelvin Probe System from KP Technology Ltd.). Calibration of the probe is done against a freshly cleaved High Oriented Pyrolytic Graphite (HOPG) surface.[10] Supplementary Figure 15a plots the Φ changes for the hybrid films on Au/mica under repeated *in situ* irradiation cycles with UV and visible light. LEDs (365 nm/ 2.68 mWcm^{-2} and 455 nm/1.34 mWcm^{-2}) connected to a channel LED driver (DC4100) from Thorlabs are used as light sources.

Upon UV light irradiation (30 s) the work function of the hybrid film drops by ~70 meV from 4.34 to 4.27 eV. This can be explained through the *trans-cis* isomerisation of the azobenzene molecules, which change their dipole upon isomerisation.[11] Φ reversibly increases upon visible light irradiation (60 s) to about the initial value. Φ can be switched over several cycles, which is a proof of the reversible *cis-trans* isomerisation. The reference samples show no Φ response to light irradiation (Supplementary Fig. 15b).

Supplementary Note 7

All molecular dynamics simulations are performed with version 4.5.5 of the GROMACS package[12] using a modified version of the OPLS-AA force field[13] developed for azobenzenes[14], in vacuum and at room temperature in the NVT ensemble[15]. The Nose-Hoover thermostat[11] is used to control the temperature. Linear Constraint Solver (LINCS) constraints are applied to all molecular bonds to restrain them to their force field equilibrium length, allowing the use of a 2fs time step. The orthorhombic simulation box measures 10 x 10 x 10 nm^3 and periodic boundary conditions (PBC) are used. A cut-off of 1.5 nm is used for both van der Waals and electrostatic interactions (plain cut-off). The SLG on which the azobenzene monolayers are modelled, is considered as an infinite rigid-body frozen in space, so to reduce the computational cost.

<u>Modelling isolated azobenzenes on graphene.</u>

Single molecules of *cis* and *trans* azobenzene adsorbed on SLG are simulated in vacuum. Since all SLG atoms are equivalent, their atomic charge is set to zero, thus the interaction between azobenzene and SLG is purely van der Waals. The molecule/SLG contact surface area is estimated by calculating the difference between the accessible solvent surface area[16] of the bare SLG and the accessible solvent surface area of the SLG with the molecules adsorbed.

<u>Modelling azobenzene self-assemblies on graphene.</u>

We first test the stability of two regular assemblies for the *trans* and *cis* molecules on a infinite SLG (Supplementary Fig. 16 a,b). The size of the orthorhombic simulation box is 16x14x10 nm, PBCs are applied and the SLG is treated as a rigid surface. Since we are interested in the stability of the assemblies, we build them smaller than the SLG, and treat them as isolated aggregates on a large SLG, so to minimize edge effects. Each monolayer contains 48 molecules and covers ~11.2 x 7.5 nm^2 for the *trans* conformation and ~9.7 x 7.5 nm^2 for *cis*. Our

simulations show that the assemblies are stable only at low temperature (150 K) while at room temperature they disassemble in a few tens of ps.

We then consider random, amorphous self-assemblies built by one molecule at a time by applying the following deposition scheme. First, the initial position of the azobenzene molecule in the simulation box is chosen as following: the X- and Y- coordinates of the center of mass are randomly chosen, while the Z-coordinate is 1.8 nm. The molecule is also randomly rotated around the X- and Y-axes between 0º and 10º and between 0º and 360º around the Z-axis. In this way each molecule is adsorbed on SLG with a different initial position and orientation, helping to form an amorphous monolayer. The molecules are placed within the cut-off distance for the interactions with graphene and are assigned initial atomic velocities vectors pointing towards SLG. A 100 ps simulation is performed in order to allow the molecule to adsorb. At the end of the simulation the deposition process is repeated and a new molecule is introduced. This procedure is iterated until the desired amount of molecules is deposited on SLG; Supplementary Fig. 17 shows some snapshots of the deposition process for the *cis* and *trans* azobenzene.

A 5ns simulation is then performed to equilibrate the system. As the deposition process does not ensure that all molecules adsorb directly on SLG, it is difficult to assess *a priori* the number of molecules needed to obtain a tightly packed self-assembled structure, therefore we deposit 80 molecules to ensure a 90% SLG coverage, regardless whether the molecules arrange in a monolayer or not (Supplementary Fig. 18).

Molecular assemblies as graphene intercalators.

We intercalate the self-assembled azobenzene structures (SAS) obtained from the deposition of 80 azobenzene molecules between two SLGs, the bottom one considered as a frozen rigid body and the upper one free to move. After 1ns at 350K, the intercalated molecules arrange in a monolayer and the distance between the two SLGs is measured. We model systems with intercalated SAS formed by 80, 72 and 64 azobenzene molecules. We find that the monolayer minimizing the graphene-graphene distance is that with 64 (72) *trans* (*cis*) azobenzenes molecules.

Electronic coupling

The system is divided into fragments, in which an electron or hole is localized on a fragment, and can hop from one fragment to another. The fragment calculations, performed by using the Zerner's Intermediate Neglect of Differential Overlap (ZINDO) method,[17] follow a

procedure where the orbitals of a pair of molecules (a dimer) are projected onto a basis set defined by the orbitals of each individual molecule (the fragments). The obtained set of orthogonal molecular orbitals energies of the dimer are then used to rewrite the Fock matrix in the new localized basis set, to obtain a block-diagonal matrix.

The main advantage of this method is the possibility to analyze pairs of different molecules, since it is not necessary to assume that the energies of the HOMO of the two fragments are equal. We apply this method to square-shape nanographenes (NG). The two molecules in the analysis are either two bare NGs or one bare NG interacting with one azobenzene-decorated NG.

Supplementary References

1. Ciesielski, A., *et al.* Liquid-Phase Exfoliation of Graphene Using Intercalating Compounds: A Supramolecular Approach. *Angew. Chem. Int. Ed.* **53**, 10355–10361 (2014).
2. Hernandez, Y., *et al.* High-yield production of graphene by liquid-phase exfoliation of graphite. *Nat. Nanotechnol.* **3**, 563-568 (2008).
3. Chen, S., *et al.* A cationic azobenzene-surfactant-modified graphene hybrid: unique photoresponse and electrochemical behavior. *Nanoscale* **7**, 19673-19686 (2015).
4. Villar-Rodil, S., Paredes, J. I., Martinez-Alonso, A., Tascon, J. M. D. Preparation of graphene dispersions and graphene-polymer composites in organic media. *J. Mat. Chem.* **19**, 3591-3593 (2009).
5. Ciesielski, A., Samorì, P. Graphene via sonication assisted liquid-phase exfoliation. *Chem. Soc. Rev.* **43**, 381-398 (2014).
6. Marago, O. M., *et al.* Brownian Motion of Graphene. *ACS Nano* **4**, 7515-7523 (2010).
7. Torrisi, F., *et al.* Inkjet-Printed Graphene Electronics. *ACS Nano* **6**, 2992-3006 (2012).
8. Baikie, I. D., Mackenzie, S., Estrup, P. J. Z., Meyer, J. A. Noise and the Kelvin Method. *Rev. Sci. Instrum.* **62**, 1326-1332 (1991).
9. Baikie, I. D., Venderbosch, E., Meyer, J. A., Estrup, P. J. Z. Analysis of Stray Capacitance in the Kelvin Method. *Rev. Sci. Instrum.* **62**, 725-735 (1991).
10. Hansen, W. N., Hansen, G. J. Standard reference surfaces for work function measurements in air. *Surf. Sci.* **481**, 172-184 (2001).
11. Kim, M., Safron, N. S., Huang, C. H., Arnold, M. S., Gopalan, P. Light-Driven Reversible Modulation of Doping in Graphene. *Nano Lett.* **12**, 182-187 (2012).
12. Abraham, M. J., *et al.* GROMACS: High performance molecular simulations through multi-level parallelism from laptops to supercomputers. *SoftwareX* **1**, 19-25 (2015).
13. Jorgensen, W. L., Tiradorives, J. The OPLS Potential Functions for Proteins - Energy Minimizations for Crystals of Cyclic-Peptides and Crambin. *J. Am. Chem. Soc.* **110**, 1657-1666 (1988).
14. Heinz, H., Vaia, R. A., Koerner, H., Farmer, B. L. Photoisomerization of Azobenzene Grafted to Layered Silicates: Simulation and Experimental Challenges. *Chem. Mater.* **20**, 6444-6456 (2008).

15. Thijssen, J. *Computational Physics*, 2 edn. Cambridge University Press: Cambridge, 2013.
16. Eisenhaber, F., Lijnzaad, P., Argos, P., Sander, C., Scharf, M. The Double Cubic Lattice Method - Efficient Approaches to Numerical-Integration of Surface-Area and Volume and to Dot Surface Contouring of Molecular Assemblies. *J. Comp. Chem.* **16**, 273-284 (1995).
17. Lipkowitz KB, Boyd DB *Reviews in Computational Chemistry*, vol. 2. Wiley-VCH, Inc.: New York, 1991.
